# Mathematical modeling of septic shock based on clinical data

**DOI:** 10.1186/s12976-019-0101-9

**Published:** 2019-03-06

**Authors:** Yukihiro Yamanaka, Kenko Uchida, Momoka Akashi, Yuta Watanabe, Arino Yaguchi, Shuji Shimamoto, Shingo Shimoda, Hitoshi Yamada, Masashi Yamashita, Hidenori Kimura

**Affiliations:** 1 Waseda University, 3-4-1 Ohkubo, Shinjuku-ku, Tokyo, Japan; 2 Tokyo Women’s Medical University, Kawada-cho, Shinjuku-ku, Tokyo, Japan; 3 Institute of Physical and Chemical Research, Moriyama-ku, Nagoya, Japan; 4 Toyota Motor Corporation, 1, Toyota-cho, Toyoda, Japan

**Keywords:** Septic shock, Model-based therapy, Blood pressure, Immune system, Inflammation

## Abstract

**Background:**

Mathematical models of diseases may provide a unified approach for establishing effective treatment strategies based on fundamental pathophysiology. However, models that are useful for clinical practice must overcome the massive complexity of human physiology and the diversity of patients’ environmental conditions. With the aim of modeling a complex disease, we choose sepsis, which is highly complex, life-threatening systemic disease with high mortality. In particular, we focused on septic shock, a subset of sepsis in which underlying circulatory and cellular/metabolic abnormalities are profound enough to substantially increase mortality. Our model includes cardiovascular, immune, nervous system models and a pharmacological model as submodels and integrates them to create a sepsis model based on pathological facts.

**Results:**

Model validation was done in two steps. First, we established a model for a *standard patient* in order to confirm the validity of our approach in general aspects. For this, we checked the correspondence between the severity of infection defined in terms of pathogen growth rate and the ease of recovery defined in terms of the intensity of treatment required for recovery. The simulations for a standard patient showed good correspondence. We then applied the same simulations to a patient with heart failure as an underlying disease. The model showed that spontaneous recovery would not occur without treatment, even for a very mild infection. This is consistent with clinical experience.

We next validated the model using clinical data of three sepsis patients. The model parameters were tuned for these patients based on the model for the standard patient used in the first part of the validation. In these cases, the simulations agreed well with clinical data. In fact, only a handful parameters need to be tuned for the simulations to match with the data.

**Conclusions:**

We have constructed a model of septic shock and have shown that it can reproduce well the time courses of treatment and disease progression. Tuning of model parameters for each patient could be easily done. This study demonstrates the feasibility of disease models, suggesting the possibility of clinical use in the prediction of disease progression, decisions on the timing of drug dosages, and the estimation of time of infection.

## Background

Sepsis is a highly complex, life-threatening systemic disease caused by infection and has a high mortality rate. The number of sepsis patients is estimated to be around 27 million per year globally, of whom approximately 8 million people die, and the number of sepsis patients is increasing [1]. The disease is sometimes referred to as *the most common but least recognized disease* [2]. In the most severe form of sepsis, called septic shock, underlying circulatory and cellular/metabolic abnormalities are profound enough to substantially increase mortality [3] and the effects of inflammation produced by the immune system spread systemically and induce an acute systemic disorder [4]. Patients with septic shock must be treated urgently in an intensive care unit. Because of its complexity, the progression of septic shock varies from patient to patient, depending on age, sex, physical characteristics, physiological activity, underlying disease, and other factors. Therefore, treatment is largely based on doctors’ skill obtained through practical experience, as is usually the case in the treatment of other diseases. Although several standard guidelines are available [5, 6], more effective, versatile, and reliable therapeutic strategies for emergency medicine are currently being sought.

The art of medicine, which emphasizes the individuality of patients, must be supported by a solid scientific understanding of disease based on human physiology. The art and science of medicine should be integrated in clinical practice at a much higher level than at present.

In the physical sciences and engineering, most of the knowledge accumulated to date about devices, components, and systems has been represented by models, most of which are presented quantitatively (mathematically). These models are available in various forms, such as scientific papers, patents, and software packages, and are used extensively as a concise representation of accumulated scientific knowledge in the research and development of new devices, components, and systems.

Accurate models of disease based on physiology and pharmacology could contribute to improving the treatment of diseases. Doctors could use such models to estimate the physiological state of their patients, predict the disease progression, and decide on treatment strategies, including the administration of drugs. Models could therefore provide a unified scientific background to clinical practice. Rami et al. extensively discussed and presented persuasive reasoning along these lines based on a historical review of treatment for sepsis [7]. They aimed to demonstrate the potential of disease models in therapy and open the door to model-based therapy.

One problem with models is to incorporate the individuality of patients. We anticipate that individual differences can be accommodated by choosing model parameters carefully based on the patients’ age, weight, sex, physiological status, underlying diseases, and other factors. Modern hospitals are well equipped with advanced diagnostic systems that would allow the easy customization of a disease model for each patient. In addition, mathematical models could help to promote a deeper understanding of diseases and establish a hypothesis of pathogenesis, improving our understanding of treatment methods.

Due to the complexity of disease physiology, it is difficult to model human diseases, and most mathematical models of disease physiology have so far focused on experimental animals, except models of diabetes and Parkinson’s disease. Treatment strategies have been developed for diabetes based on mathematical models [8]. The model developed by Kovatchev et al. [9] was approved by the US Federal Drug Administration as an alternative to animal research for the approval of diabetes medications. Recently, we have constructed a diabetes model that includes brain-centered glucose metabolism and suggested an alternative therapeutic strategy for diabetes [10]. A mathematical model based on brain metabolism has been constructed for Parkinson’s disease and is recognized as a useful tool for investigating its pathogenesis [11, 12]. The importance of mathematical models in understanding the basic physiology in the progression of sepsis has been highlighted in previous work [13]. In addition, Kendrick et al. published a clear description of the immune response to sepsis [14], and Shi et al. discussed a bifurcation analysis of sepsis based on an immune system model [15].

In this study, we aimed to construct a new mathematical model of septic shock based on clinical data. Among the diverse symptoms of sepsis patients, we focused on the damage caused to the cardiovascular system because septic shock most frequently damages the cardiovascular system. Our model combines the cardiovascular system, immune system, and pharmacological models, and we used existing models of these systems as our guiding tools [16, 17]. We focused on how inflammation resulting from immune activity affected the cardiovascular system and caused septic shock. Among the many possible effects of inflammation on the cardiovascular system, we selected increased vessel permeability, vasodilation, and reduced stroke volume [18, 19]. We incorporated these three factors into the combined model of the cardiovascular and immune systems, making the resulting model highly nonlinear. Through simulations, we showed that these three factors are sufficient to reproduce septic shock.

To complete the sepsis model, the nervous system and pharmacological responses to drug administration must be incorporated because they are crucial to the disease model. We could not measure the activity of the nervous system, but we incorporated qualitative physiological and empirical data to achieve a quantitative description in our model to reflect realistic physiological effects. The activity of the nervous system is weaker in patients with sepsis than in healthy people; thus, we introduced fatigue as a parameter of the sympathetic nervous system [20, 21]. In addition, the effects of drugs are reduced in sepsis patients compared with healthy people. Therefore, we used experimental data showing the reduced effects of an antihypertensive medicine in sepsis patients.

## Method

**Fig. 1:**
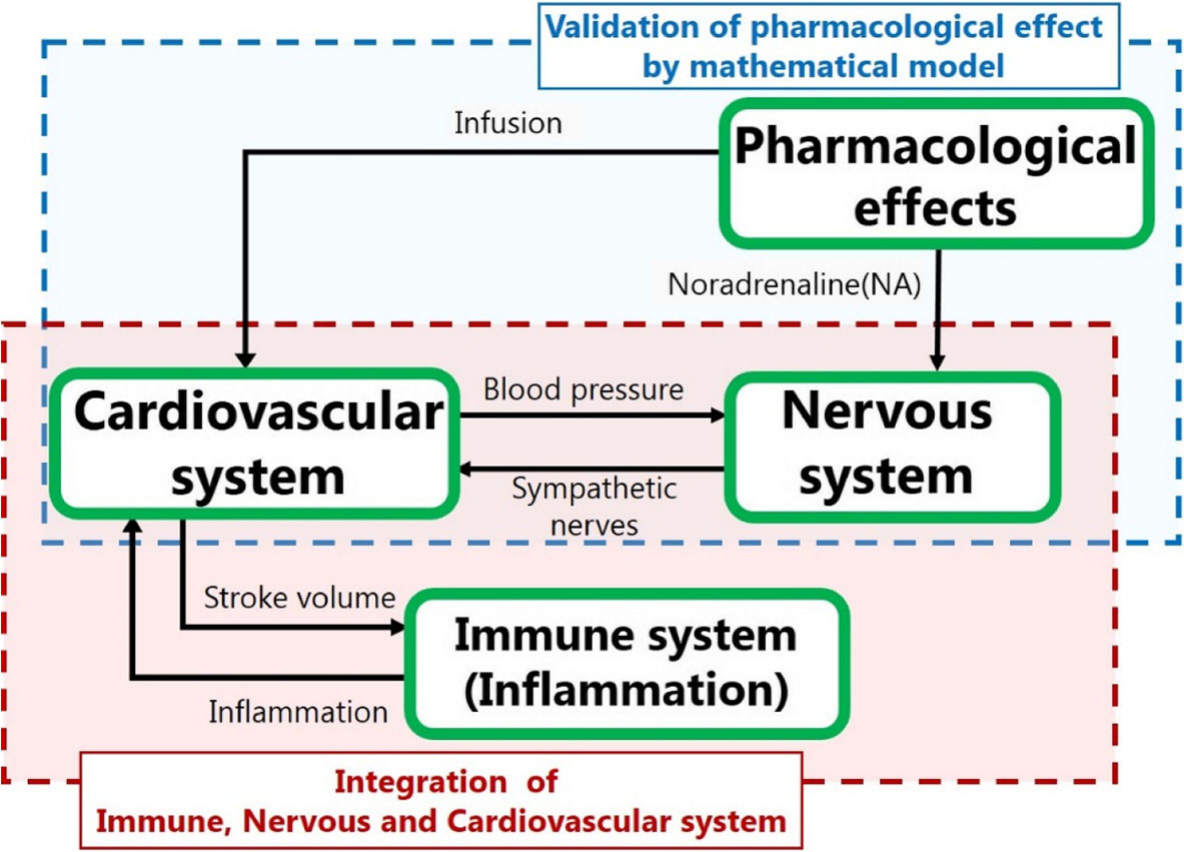
Overview of the sepsis model

We constructed a mathematical model that represents the physiological dynamics of septic shock after infection and comprises cardiovascular system, immune system, nervous system, and pharmacological submodels. An overview of our model is shown in Fig. 1. There are various cardiovascular, nervous, and immune system models for different uses in the literature. Most of these models are closed in the single-target domain, although they must be connected to represent the disease. In this study, we focused on integrating these models, based on choosing appropriate existing models for the sepsis model. We used the cardiovascular system model proposed by Ursino and Innocenti [17], which is comprehensive and includes the solute kinetics of each constituent in blood, as well as the sympathetic nervous system. Because the increase in vascular permeability is an important effect of inflammation on the cardiovascular system, the solute kinetics of the systemic capillaries in the model are essential in our sepsis model.

The immune system is complex, and quantitative models are still incomplete [22, 23]. We based our sepsis model on the model reported by Reynolds et al. [16] because it is simple but captures the essential features of the immune system that are relevant to our sepsis model. We incorporated the effect of antibiotics into this model, following the proposal of Kitamura [24].

The core of our sepsis model is in the link between the cardiovascular and immune systems. In other words, we model how inflammatory responses damage the cardiovascular system. As stated in the Background section, we considered the three effects of inflammation on the cardiovascular system—increased vessel permeability, vasodilation, and reduced stroke volume—all of which contribute to reducing blood pressure. To quantify these effects, we represented the three parameters as functions of inflammation. Because inflammation manifests in diverse ways, it is hard to represent as a simple physical quantity; it is more an abstract and collective quantity. In contrast, permeability, vasodilation, and stroke volume are tangible physical parameters with clear units of measurement. The model connected these physical parameters with an abstract representation of the severity of inflammation. This was an unavoidable difficulty and an intriguing aspect of sepsis modeling.

Next, we briefly describe each model.

**Fig. 2:**
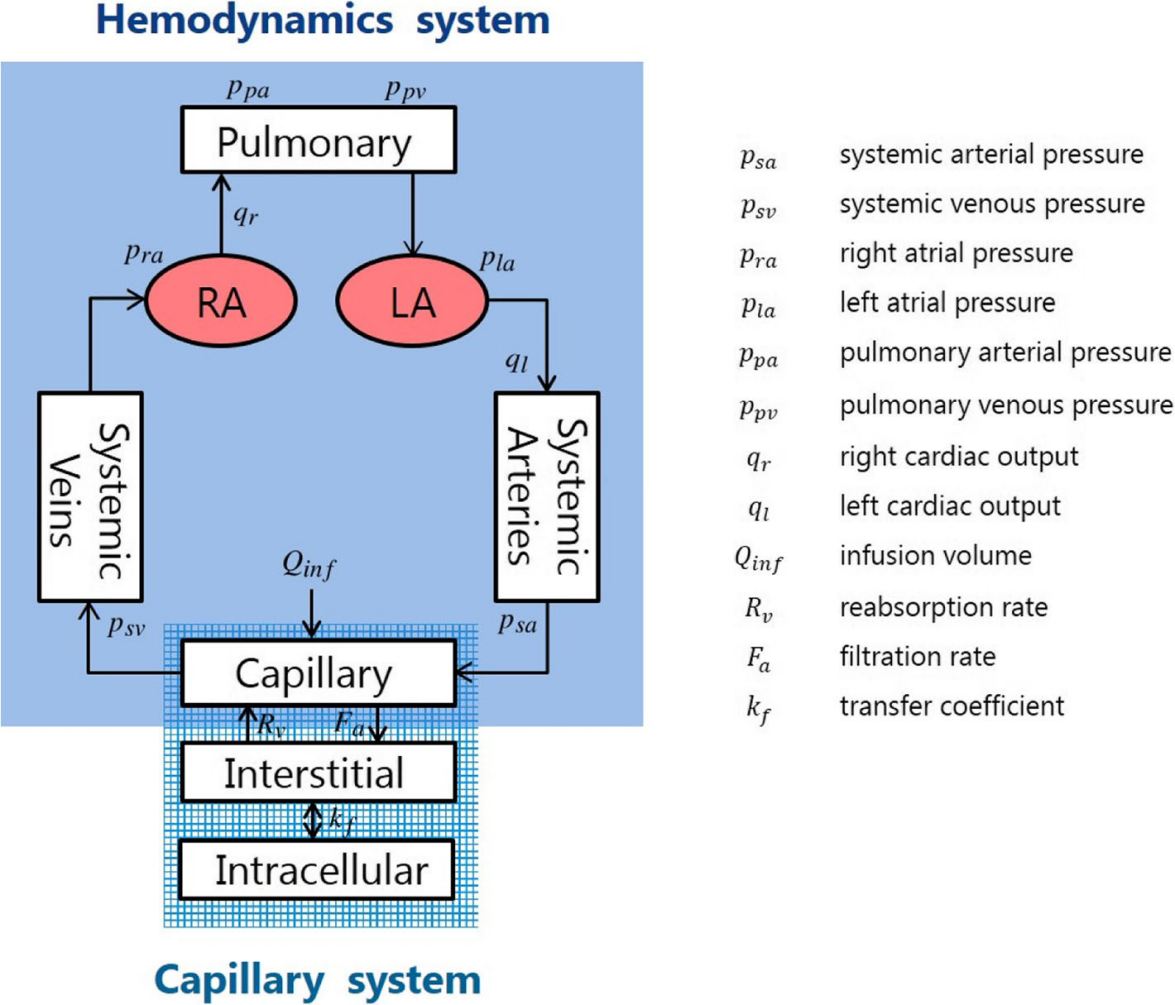
Cardiovascular system model

The cardiovascular system model is composed of five compartments, namely, the pulmonary atrium (*pa*), right atrium (*ra*)*,* left atrium (*la*), systemic arteries (*sa*), and the systemic veins (*sv*) (Fig. 2).

Each compartment is described by its volume *V*, pressure *P*, incoming flow rate *q*_*in*_, outgoing flow rate *q*_*out*_, and compliance *C* representing the compartment capacity, subject to the conservation of mass

1 $$ \frac{dV}{dt}={q}_{in}-{q}_{out}, $$

2 $$ V= CP $$

The right cardiac output *q*_*r*_ and left cardiac output *q*_*l*_ are represented by.

3 $$ {q}_r={S}_r\;f,\kern0.5em {q}_l={S}_l\;f, $$

where *S*_*r*_ and *S*_*l*_ are the right and left stroke volumes, respectively, and *f* is the heart rate.

The solute kinetics of the capillary system that transports the blood components to the tissues are important in our model. We focus on the material exchange between vessels and the interstitial fluid. The total blood volume *V* is subject to the following transport law:

4 $$ \frac{dV}{dt}=-{F}_a+{R}_v+{Q}_{inf} $$

where *Q*_*inf*_ denotes the external infusion rate. Outflow *F*_*a*_ from the vessel to the interstitial space and inflow *R*_*v*_ in the opposite direction in eq. (4) are determined by blood pressure and oncotic pressure as

5 $$ {F}_a={L}_a\left\{\left({P}_{ac}-{P}_{is}\right)-\left({\pi}_{pl}-{\pi}_{is}\right)\right\} $$

6 $$ {R}_v={L}_v\left\{\left({P}_{is}-{P}_{vc}\right)-\left({\pi}_{is}-{\pi}_{pl}\right)\right\} $$

where *P*_ac_ is the capillary arterial pressure, *P*_*is*_ the interstitial fluid pressure, *P*_*vc*_ the venous capillary pressure, *π*_*pl*_ the plasma oncotic pressure, and *π*_*is*_ the interstitial oncotic pressure. Coefficient *L*_*a*_ in equation (5) denotes vessel permeability, which is important in our model, whereas coefficient *L*_*v*_ in equation (6) denotes another permeability characterizing the opposite blood flow, which is considered to be irrelevant to damage. In reality, there are more inputs and outputs that affect the total blood volume, such as the blood carried to the kidneys. However, we neglected these other factors because their contributions are relatively small. The more detailed solute dynamics associated with equations (5) and (6) are described by Ursino and Innocenti [17].

Total blood volume *V* consists of six components, namely,

7 $$ V={V}_u+{V}_f,\kern0.5em {V}_f={V}_{\rho a}+{V}_{ra}+{V}_{la}+{V}_{sa}+{V}_{sv} $$

where *V*_*u*_ is the unloaded volume and *V*_*f*_ is the filling volume which consists of the volume in each compartment. The unloaded volume is the part of blood reservoir in the heart that does not circulate.

The baroreflex is governed by the sympathetic nervous system, which elevates the blood pressure when the baroreceptors detect a decrease in blood pressure. The increase in blood pressure is achieved via elevation of the heart rate, increased vascular resistance, and increased venous blood volume unloading [25].

Let *a* be the action of the sympathetic nervous system. The mechanisms of nervous system action are different for each component of the baroreflex; thus, *a* for heart rate elevation is denoted by *a*_*f*_, *a* for increasing vascular resistance is denoted by *a*_*r*_, and *a* for increasing unloaded blood volume is denoted by *a*_*υ*_. The elevation in heart rate mediated by sympathetic action is described as

8 $$ f={f}_0\left(1-{a}_{0f}+{a}_f\right) $$

where *f*_0_ is the normal heart rate and *a*_0*f*_ is the normal level of sympathetic nervous system activity.

According to Poiseuille’s law, vessel resistance *R* is inversely proportional to the fourth power of the vessel radius *r*, that is,

9 $$ R\frac{Q}{r^4}={r}_0{K}_{r, cr}, $$

where *r*_0_ is the normal vessel radius, $Q/{r}_o^4$ is the normal vessel resistance, and *K*_*r*, *cr*_ represents the change in the vessel radius due to sympathetic nerve activity *a*_*r*_. We assume that sympathetic nerve activity decreases the vessel radius as

10 $$ {K}_{r, cr}=\frac{1}{1-{a}_{0r}+{a}_r}=\frac{1}{A_r} $$

If *a*_*r*_ increases above *a*_0*r*_, then *K*_*r*, *cr*_ and resistance *R* decrease.

Finally, the unloaded blood volume *V*_*u*_ in equation (7) is assumed to be reduced by the sympathetic nervous system in the same way as in equation (10),

11 $$ {V}_u={V}_{u0}{K}_{v, cr},\kern0.5em {K}_{V, cr}=\frac{1}{1-{a}_{0v}+{a}_v}=\frac{1}{A_V}, $$

where *V*_*u*0_ is the normal unloaded blood volume *V*_*u*_ given in equation (7) [17]. The reduction of the unloaded volume implies an increase in circulating volume *V*_*f*_ due to equation (5), assuming that total volume *V* is fixed.

Now we quantify the baroreflex and its fatigue. Let *X* be the average output of the baroreceptors that detect the right arterial blood pressure, *P*_*ra*_, and the systemic arterial blood pressure, *P*_*sa*_, which is assumed to be

12 $$ X={q}_r{P}_{ra}+{q}_s{P}_{sa} $$

where *q*_*r*_ and *q*_*s*_ are averaging factors. The sympathetic nervous system responds to the decreasing pressure signal represented by

13 $$ \Delta  \mathrm{X}={X}_0-X $$

where *X*_0_ is the normal baroreceptor signal given by.

14 $$ {X}_0={q}_r{P}_{ra0}+{q}_s{P}_{sa0} $$

The normal arterial blood pressure, *P*_*ra*0_, and systemic blood pressure, *P*_*sa*0_, depend on individual patients.

Since the nerve activities *a*_*f*_, *a*_*r*_ and *a*_*υ*_ have the same mathematical representations, we omit their subscripts f, r, and υ in the following description. We assume that *a* changes between its minimum, *a*_*min*_, and maximum, *a*_*max*_, due to a change in *X*. Thus, *a* is assumed to be represented by a sigmoid function of *∆X*:

15 $$ a=\frac{a_{max}-{a}_{min}}{1+\mathit{\exp}\left(-\Delta  X/{X}_0\right)}+{a}_{min} $$

The normal level of sympathetic nerve activity *a*_0_ corresponds to the activity level when *∆X* = 0. Hence, equation (15) implies

16 $$ {a}_0=\frac{a_{max}+{a}_{min}}{2} $$

that is, the average of *a*_*max*_ and *a*_*min*_.

If sympathetic nerve activity is sustained above its normal level for a long time, then the action gradually decreases due to fatigue (e.g., [20]). To represent this effect, we introduce fatigue factor γ as

17 $$ \dot{\gamma}=\frac{1}{\tau_{\gamma }}\left(a-{a}_0\right) $$

and γ decreases the nervous activity as

18 $$ a=\frac{a_{max}-{a}_{min}}{1+\mathit{\exp}\left(-\Delta  X/{X}_0+\gamma \right)}+{a}_{min} $$

If *a*_0_ < *a* for an extended time, γ increases and *a* is reduced according to equation (18). Equations (17) and (16) are nonlinear differential equations.

Sepsis is caused by excessive inflammation triggered by the immune system after infection. The dynamics of the immune system play an important role in evaluating the progression of sepsis. However, because the immune system is complex, mathematical models of immune system dynamics are not well developed, although there have been several attempts to quantify the dynamics [23, 25]. We base our sepsis model on the model proposed by Reynolds et al. [16] because their model is simple but captures some essential features of the immune system that are relevant to sepsis.

**Fig. 3:**
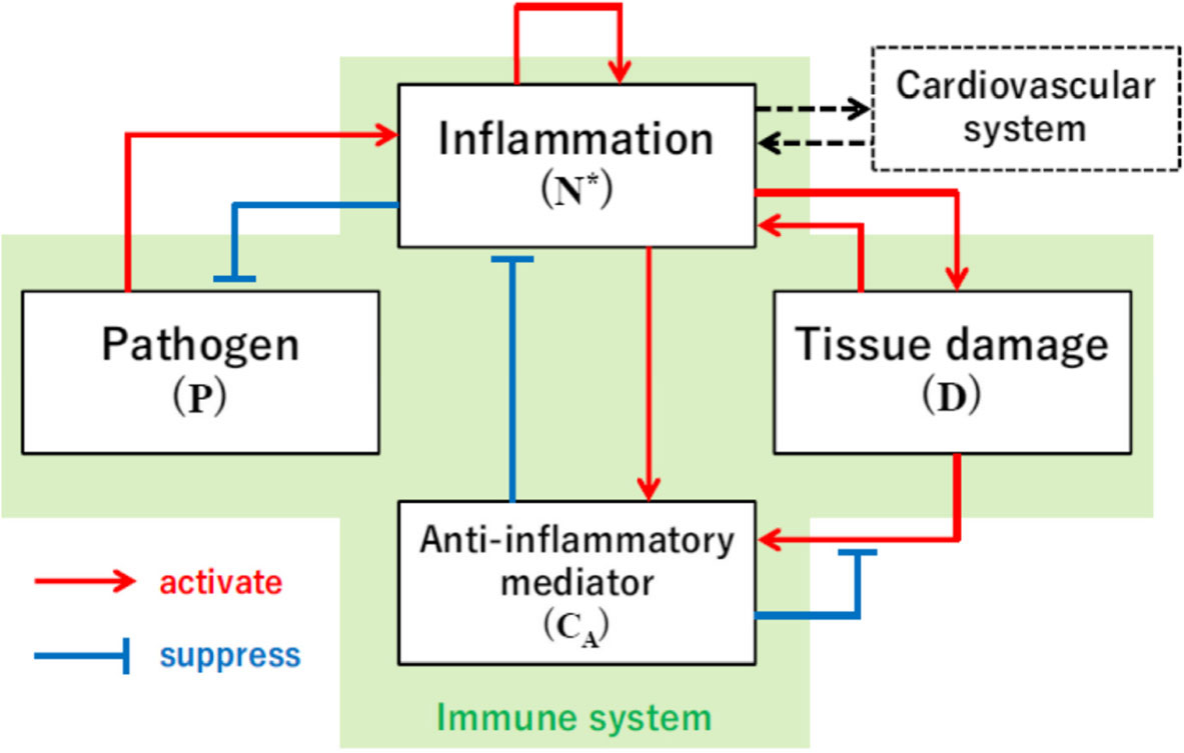
Overview of the immune system model [16]

The dynamic model is composed of the four state variables, pathogen population *P*, inflammation *N*^∗^, damage *D*, and anti-inflammatory mediator *C*_*A*_ (Fig. 3) [16].

The interactions among these variables are described as follows.

The dynamics of *P* are described by.

19 $$ \frac{dP}{dt}={k}_{pg}P\left(1-\frac{P}{P\infty}\right)-\frac{k_{pm}{S}_mP}{\mu_m+{k}_{mp}P}-{k}_{pn}g\left({N}^{\ast}\right)P-\varepsilon \frac{C_f^{\gamma }}{C_f^{\gamma }+{EC}_{50}^{\gamma }},P $$

20 $$ g(x)=\frac{x}{1+{\left({C}_A/{C}_{\infty}\right)}^2} $$

The first term represents the logistic growth of pathogen *P*, where *k*_*pg*_ is the growth rate and *P*_∞_ is the carrying capacity of *P*. The second term represents the non-specific local immune response toward *P* characterized by the Michaelis–Menten equation [16]. The third term represents the removal of the pathogen by phagocytic immune cells, which is proportional to inflammation *N*^∗^, restricted by the anti-inflammatory mediator *C*_*A*_, as shown in equation (20). The forth term represents the effect of antibiotic dosage proposed by Kitamura [24]. Here, *C*_*f*_ denotes the free concentration of antibiotic, which is subject to the following dynamics.

21 $$ \frac{d{X}_1}{dt}=-{k}_a{X}_1 $$

22 $$ \frac{d{X}_2}{dt}={k}_a{X}_1-{k}_e{X}_2 $$

23 $$ {C}_f={f}_p{X}_2/{V}_d $$

Here, *X*_1_ denotes antibiotic dosage, *X*_2_ its blood concentration, *k*_*a*_ the absorption coefficient and *k*_*e*_ the degradation coefficient.

The inflammation dynamics are represented by

24 $$ \frac{dN^{\ast }}{dt}=\frac{S_{nr}R}{\mu_{nr}+R}-{\mu}_n{N}^{\ast } $$

25 $$ \mathrm{R}=g\left({k}_{nn}{N}^{\ast }+{k}_{np}P+{k}_{nd}D\right) $$

The first term of equation (24) is a simplified representation of the initiation of inflammation caused by *P*, *D*, and *N*^∗^ represented by their linear combination in equation (25), and *s*_*nr*_ and *μ*_*nr*_ are Michaelis–Menten parameters for the inflammatory reactions. Function *g* introduced in equation (20), which represents the saturating factor due to the presence of anti-inflammatory mediator *C*_*A*_, is also used to represent the initiation of inflammation. The second term represents the degradation.

*C*_*A*_ is subject to the dynamics

26 $$ \frac{d{C}_A}{d^t}={S}_c+\frac{k_{cn}g\left({N}^{\ast }+{k}_{cn d}D\right)}{1+g\left({N}^{\ast }+{k}_{cn d}D\right)}-{\mu}_C{C}_A $$

Here, *S*_*c*_ denotes a source of *C*_*A*_ and the second term represents the production of *C*_*A*_ from damage *D* and inflammation *N*^∗^ by a Michaelis–Menten term with inhibition mediated by *C*_*A*_ itself. The third term represents the degradation. More detailed descriptions are found in Reynolds et al. [16].

Damage *D* is an abstract quantity in the paper by Reynolds et al. [16], but here we give it a physical meaning to represent cardiovascular system damage. There are several ways to identify cardiovascular damage, and we take reduced stroke volume *S*_*l*_, introduced in equation (3), because it affects the whole system substantially. We describe the damage as

27 $$ \frac{dD}{dt}={k}_D{S}_d-{\mu}_dD $$

28 $$ {S}_d=\left\{\begin{array}{cc}\frac{1}{1+{e}^{\left(k{S}_l/{S}_0-{k}_0\right)}},& {S}_l\le {S}_0\\ {}0& {S}_l>{S}_0\end{array}\right. $$

where *S*_0_ is the normal stroke volume and *S*_*d*_ is a decreasing sigmoid function that takes the value 1/(1 + *e*^−*k*0^) (i.e., approximately 1) when *S*_*l*_ = 0, and $1/\left(1+{e}^{\left(k-{k}_0\right)}\right)$ (i.e., approximately 0) when *S*_*l*_ = *S*_0_, provided that appropriate values of *k* and *k*_0_ are used.

Next, we quantify how inflammation lowers blood pressure. The most important factor is the increase in the permeability of the capillaries due to inflammation [16, 18]. In our model, capillary permeability is represented by coefficient *L*_*a*_ in equation (5). We assume that the inflammation population *N*^∗^ increases *L*_*a*_ following a sigmoid function given by

29 $$ {L}_a=\frac{L_{a,\mathit{\max}}-{L}_{a,\mathit{\min}}}{1+{\left({EC}_{50, La}/{N}^{\ast}\right)}^{slopeLa}}+{L}_{a,\mathit{\min}} $$

where *L*_*a*, *max*_ and *L*_*a*, *min*_ are the maximum and minimum levels of permeability, respectively. Equation (29) is in the same form as equation (18). If *N*^∗^ is large, *L*_*a*_ goes to *L*_*a*, *max*_, whereas if *N*^∗^ is negligibly small, *L*_*a*_ becomes equal to *L*_*a*, *min*_.

The vessel radius *r* is given by equation (9), and we assume *A*_*r*_ in (10), which represents the dilation factor, *K*_*r*, *cr*_, is now reduced by *N*^∗^ as

30 $$ {A}_r\to {A}_r- EX,\kern0.5em EX=\frac{k_{EX}}{1+{\left({EC}_{50, EX}/{N}^{\ast}\right)}^{slopeEX}} $$

to represent the effect of inflammation. *EX* tends to zero as *N*^∗^ approaches zero.

Finally, we assume that inflammation damages the function of the heart substantially [13, 23]. We assume that inflammation decreases the left stroke volume *S*_*l*_ defined by equation (3) as

31 $$ {S}_l=\frac{S_0}{1+{k}_sg\left({N}^{\ast}\right)} $$

where *S*_0_ denotes the normal stroke volume and *g* is given by equation (20).

Lowered blood pressure in septic shock is treated by infusion and drugs. Infusion is represented by the term *Q*_*inf*_ in equation (4). An infusion may contain many blood components and varies according to the condition of the patient. However, we omitted a detailed description of the components and assume the infusion to be 0.9% saline.

There are several drugs used to treat severe hypotension in sepsis patients, of which noradrenaline and dopamine are the most commonly used in clinical practice. Antibiotics are also used to dispose the pathogen and are represented by the fourth term of equation (19).

**Fig. 4:**
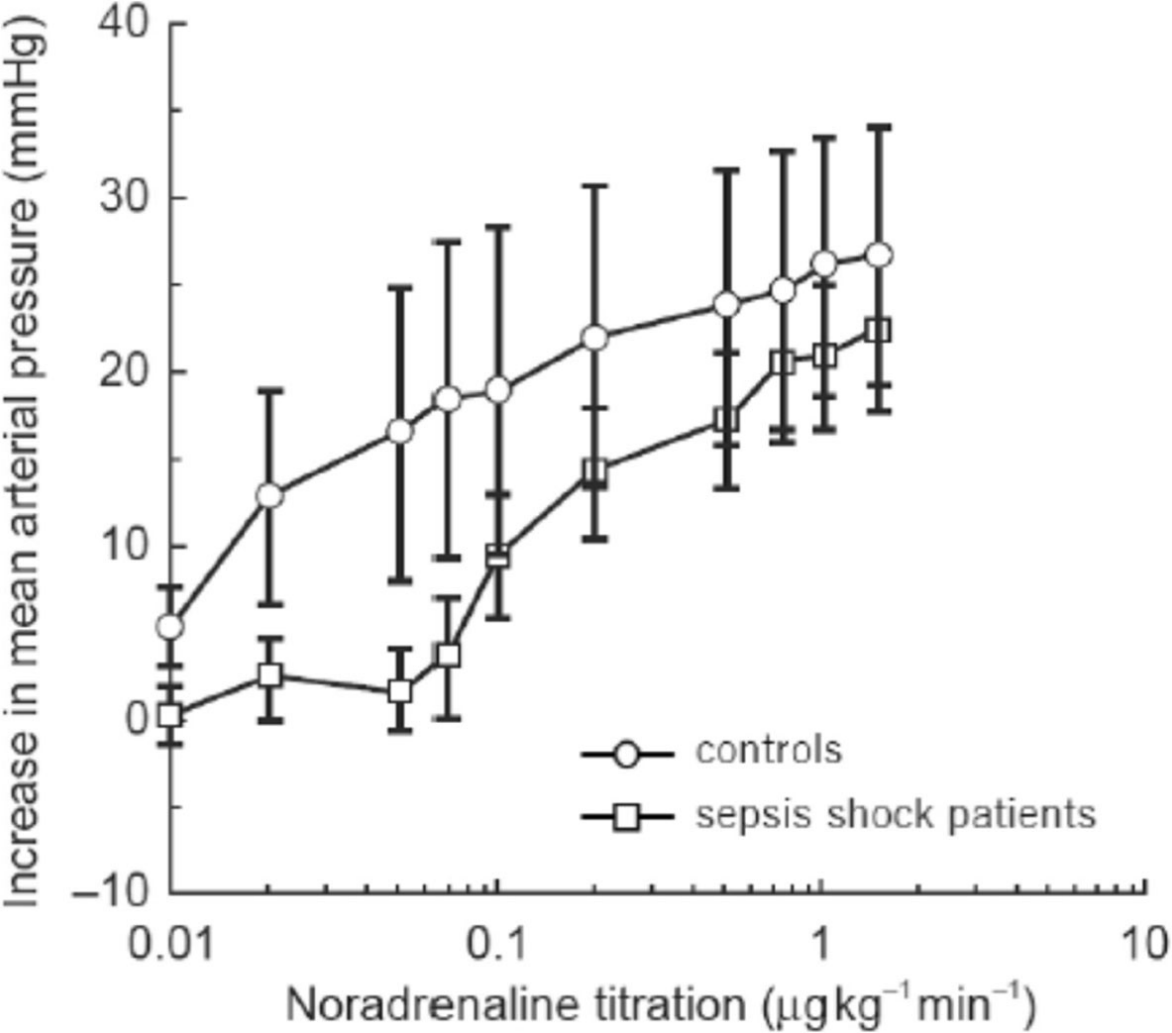
Experimental results of the effects of noradrenaline on mean arterial pressure. [27]

**Fig. 5:**
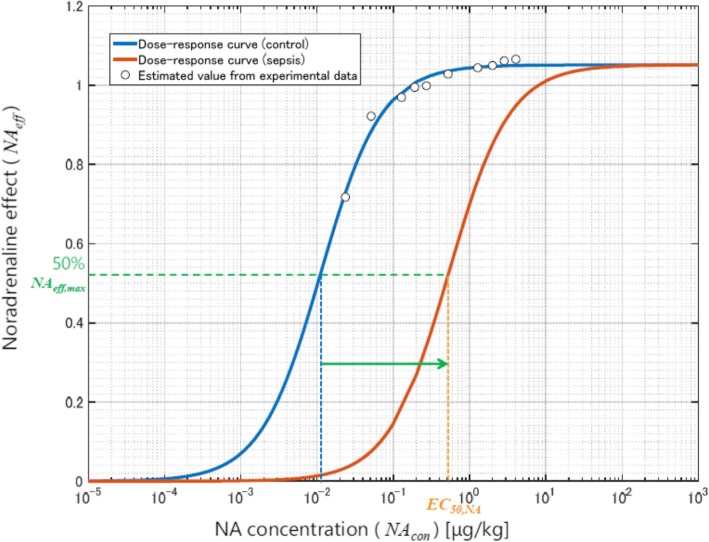
Noradrenaline effect model (dose-response curve)

The dose-response curve of noradrenaline (*NA*_eff_) is normally represented by a sigmoid function

32 $$ {NA}_{eff}=\frac{NA_{eff.\mathit{\max}}}{1+{\left({NA}_c/{EC}_{50, NA}\right)}^{- slopeNA}} $$

where *NA*_*c*_ denotes the concentration of noradrenaline in the vessels. Although the noradrenaline dose-response curve is available for healthy individuals [25], it cannot be applied for patients with sepsis because the effect of noradrenaline is weaker in these patients than in healthy people [26–28]. The effect of noradrenaline in the treatment of hypotension in patients with sepsis compared with healthy controls is shown in Fig. 4, which was reproduced from the paper by Annane D. et.al. [27]. The reduction in the effect is significant and should be considered in models of sepsis treatment. We tuned parameters *EC*_50, *NA*_ and *slopeNA* in equation (32) to fit the clinical data of noradrenaline administration to the controls of Fig. 4. We noticed that the clinical data in Fig. 4 for sepsis patients could be reproduced by simply increasing *EC*_50, *NA*_ by a factor of approximately 10^2^ (Fig. 5).

Noradrenaline acts in various ways to elevate blood pressure. Here, we simply assumed that noradrenaline increases the effect of sympathetic nerve activity *a*. Thus, after the dosage *NA*_*in*_, sympathetic nerve activity is assumed to be increased by a factor proportional to *NA*_*eff*_, that is,

33 $$ a\to a+{kNA}_{eff} $$

where *k* is a coefficient representing the reinforcing effect of noradrenaline. In the computation, *a* is changed to *a* + *kNA*_*eff*_ after the dosage of *NA* wherever *a* appears.

Another major drug for sepsis therapy is dopamine, the main effects of which are increasing the heart rate and stroke volume [26]. These effects are described as

34 $$ {f}^{\prime }=f\left(1+{G}_{D,f}\bullet DO\right) $$

35 $$ {S}_l^{\prime }={S}_l\left(1+{G}_{D,s}\bullet DO\right) $$

where *f* and *S*_*l*_ are heart rate and stroke volume, respectively, *DO* is the dopamine concentration, and *G*_*D*, *f*_ and *G*_*D*, *s*_ are the coefficients of the effects of dopamine on *f* and *S*_*l*_, respectively. Here, we assume that *f* and *S*_*l*_ are increased to *f*^′^ and ${S}_l^{\prime }$, respectively, due to the dopamine dosage. We assume that dopamine becomes effective through the first-order transfer process,

36 $$ {T}_D\frac{dD}{dt}={DO}_{in}- DO $$

where *DO*_*in*_ denotes the actual dosage of dopamine.

### Parameters

**Fig. 6:**
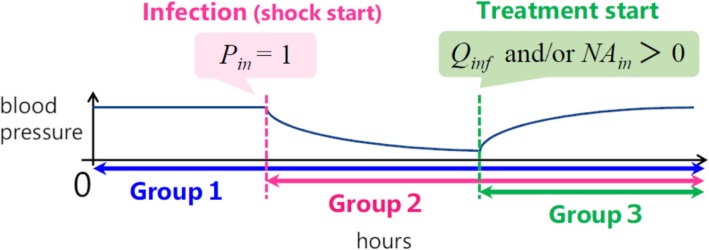
Parameter classification

Our model contained a number of parameters that must be quantified to perform simulations. We classified them into three groups according to the time periods in which they were used (Fig. 6).

The parameters in group 1 are used throughout the whole simulation, even before infection. They represent the physical characteristics of the patient, such as weight, sex, and underlying diseases. All parameters in the cardiovascular system are taken from the paper by Ursino and Innocenti [17]. Their numerical values are shown in Table A1 of the Appendix.

The parameters related to the nervous system used in equations (9)–(18) are shown in Table A2 in the Appendix. Some of them are taken from Ursino and Innocenti’s paper [17], and others are estimated mainly based on the literature. Some parameters are fitted based on MATLAB tools to minimize the gap between data and simulation. .

The initial value of the total blood volume *V* depends on sex and weight. We assume that the total blood volume is 8% of body weight for men and 7% for women [29]. We also consider the possibility of heart failure as an underlying disease. The quantitative description of heart failure is presented in the Results section.

The parameters in group 2 are used after the initiation of infection and include the immune system parameters. We take these parameters from the paper by Reynolds et al. [16] (Table A3). This group also contains parameters that represent the effects of infection on the cardiovascular system. The most important parameters in this group are those that represent the increase in blood permeability *L*_*a*_ denoted by equation (5). There are several papers that report attempts to measure blood permeability. It was reported that the maximum value of *L*_*a*_ during infection is almost 6 times greater than normal *L*_*a*_ [30–32], and we used this observation in our model. The other parameters in equation (29) are chosen by tuning and are listed in Table A4 in the Appendix.

The growth rate of the pathogen, given by parameter *k*_*pg*_, is used to represent the severity of the infection. Other parameters are taken from the model reported by Reynolds et al. [16].

The parameters in group 3 are pharmacological parameters that represent drug efficacy [24]. The dose-response curve of noradrenaline is represented by sigmoid function in equation (32) and the numerical values of the associated parameters have been experimentally obtained for healthy subjects [25]. The dose-response curve for sepsis patients may differ from that for healthy people. The numerical values of the parameters in equation (30) are listed in Table A5.

## Results

We validated the model in two steps. In the first step, we established *a standard patient model* capturing some essential features of sepsis progression and treatment effect, at least qualitatively. For this purpose, the relationship between the severity of the infection and the difficulty of recovery was important in the disease model. We represented the severity of infection through the value of parameter *k*_*pg*_ in equation (19), which describes the growth rate of the pathogen.

We took heart failure as a representative example of an underlying disease in patients due to the strong link between sepsis and cardiac insufficiency [33, 34]. A typical consequence of heart failure is a reduction in stroke volume. According to the European Society of Cardiology guidelines published in 2016 [34], heart failure is defined as a circulatory condition in which the ejection fraction (EF) is below 40%, where EF is defined as the ratio of the left heart cardiac stroke volume to the left heart blood volume. Normal EF is between 50 and 60%. We noticed that if the left cardiac stroke volume *S*_*l*_ in equation (3) was reduced by 22%, we obtained a 40% drop in EF, which is consistent with the definition of heart failure with reduced EF. Therefore, we used this reduction in *S*_*l*_ to represent heart failure as the underlying disease.

We classified the severity of infection as mild, moderate, and severe, based on the range of parameter *k*_*pg*_. Sepsis progression was represented in the time courses of mean arterial pressure (MAP) and heart rate. The recovery can be judged when the time course of MAP and heart rate returned to the normal or original level.

**Fig. 7:**
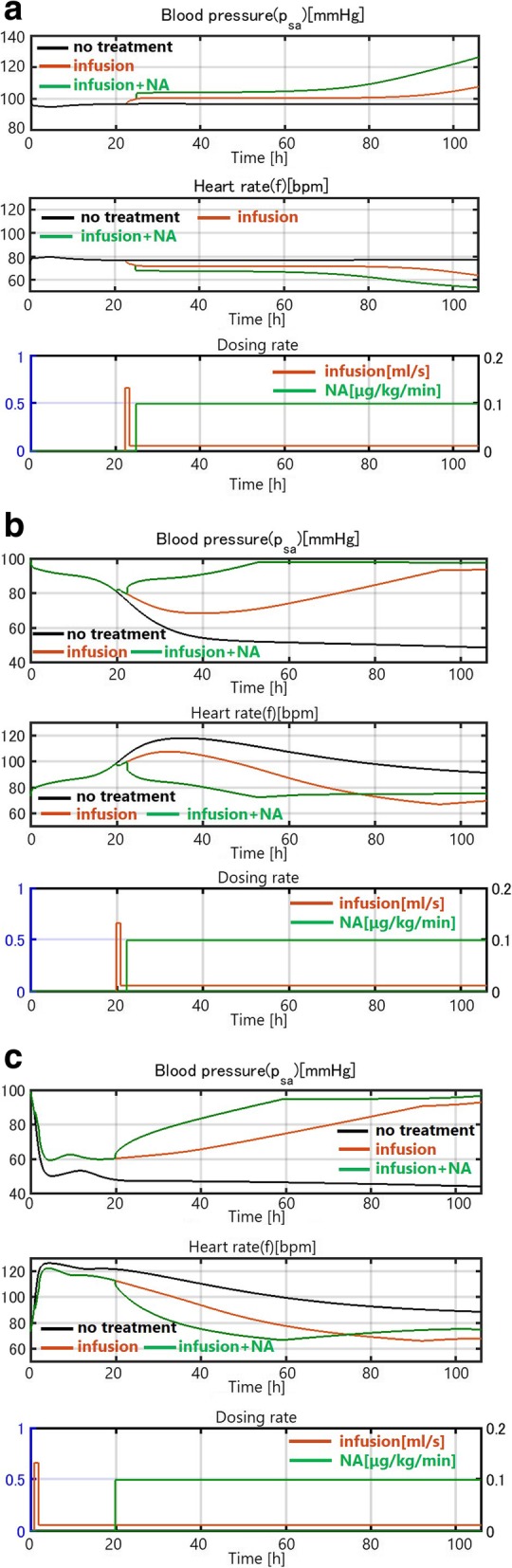
(**a**) Patient with a mild infection (*k*_*pg*_ = 0.2). (**b**) Patient with a moderate infection (*k*_*pg*_ = 0.45). (**c**) Patient with a severe infection (*k*_*pg*_ = 1.50). Time courses of sepsis development

For mild infection, where *k*_*pg*_ is small (*k*_*pg*_ = 0.2), the disease spontaneously resolves without treatment (black curve). The internal immune system works effectively, although the blood pressure decreases slightly and temporarily (Fig. 7(a)). Thus, natural healing due to the innate immune system is achieved. An additional simulation shows that saline infusion (red curve) and noradrenaline (green curve) improve the recovery process in the mild infection case (Fig. 7(a)).

For moderate infection (*k*_*pg*_ = 0.45), the internal immune system alone cannot control the effect of inflammation and the blood pressure continues to decrease (Fig. 7(b)). However, infusion can prevent the decrease in blood pressure. Blood pressure does not decrease even after the infusion rate is reduced to the minimum level after 1 h of intensive infusion, which is consistent with clinical data.

For severe infection with a high *k*_*pg*_ (*k*_*pg*_ = 1.50), infusion alone is not enough to raise the blood pressure and noradrenaline is needed (Fig. 7(c)).

**Fig. 8:**
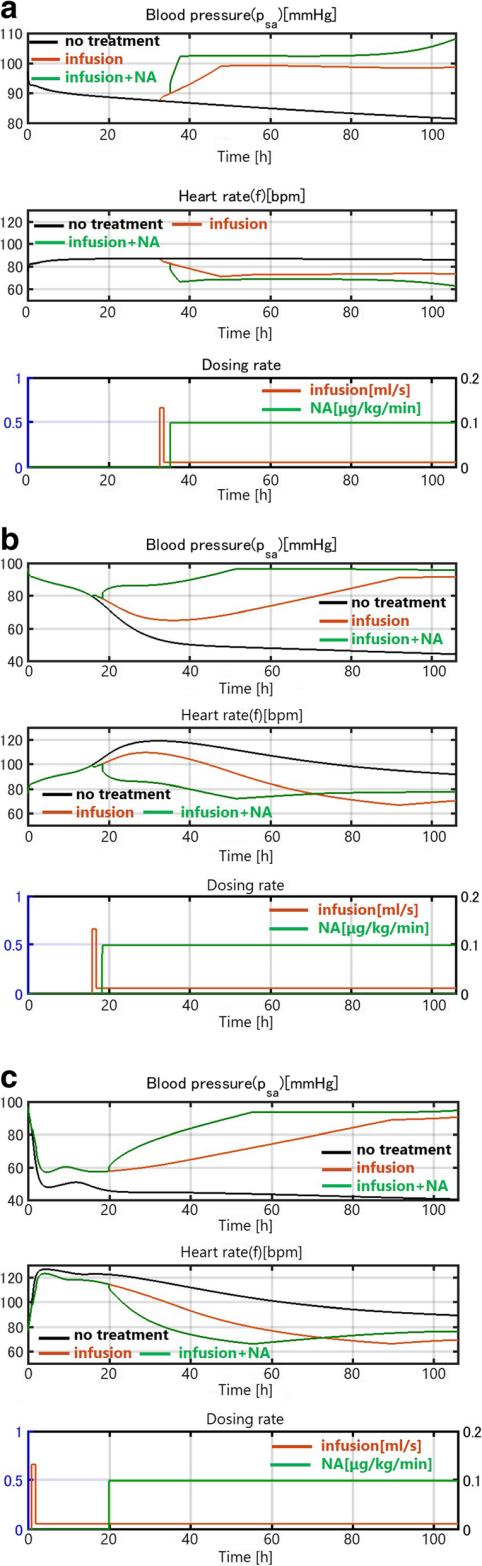
(**a**) Mild infection with heart failure. (**b**) Moderate infection with heart failure. (**c**) Severe infection with heart failure. Time courses of sepsis development in patients with heart failure

We conducted the same simulations for a case with heart failure as an underlying disease. In this patient, no spontaneous healing occurred. Even in the case of mild infection, the blood pressure continued to drop without treatment, as shown in Fig. 8(a) (black curve). This is consistent with clinical observations that heart failure often seriously affects sepsis progression. An infusion can resolve the drop in0020blood pressure, as in patients without heart failure. The time courses in Figs. 7(b)(c) and 8(b)(c) are similar, indicating that in cases of moderate and severe infection, the sepsis damage dominates the effect of heart failure as an underlying disease, which is also consistent with some clinical observations.

The two in silico experiments show that our model reproduced the progression of septic shock and the outcome of the treatment, at least qualitatively.

Now, we validated our model quantitatively using real clinical data from three patients with septic shock who were treated in Tokyo Women’s Medical University Hospital. Basic information about the patients is given in Table 1.

**Table 1:** Basic Information about Patients

	Patient’s Data				Treatment			
	Sex	Age	Weight[kg]	Underlying Disease	Infusion	NA	DOA	Antibiotics
Patient1	male	51	66	Heart failure*	○	○	×	○
Patient2	female	99	45	Alzheimer	○	×	○	×
Patient3	male	79	65	Diabetes Atrial fibrillation	○	○	×	×
								*assumed

**Fig. 9:**
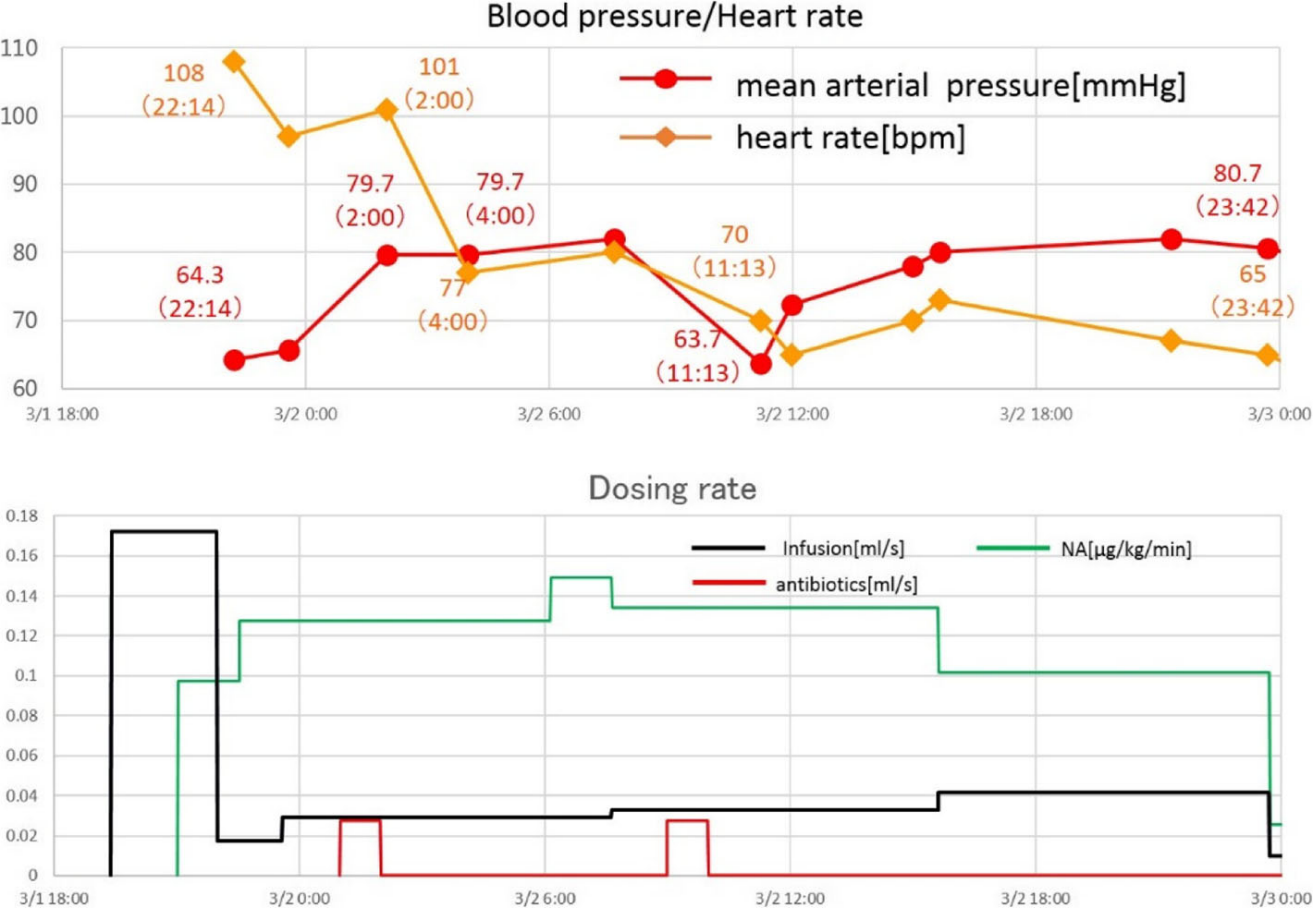
Clinical data for patient 1

**Fig. 10:**
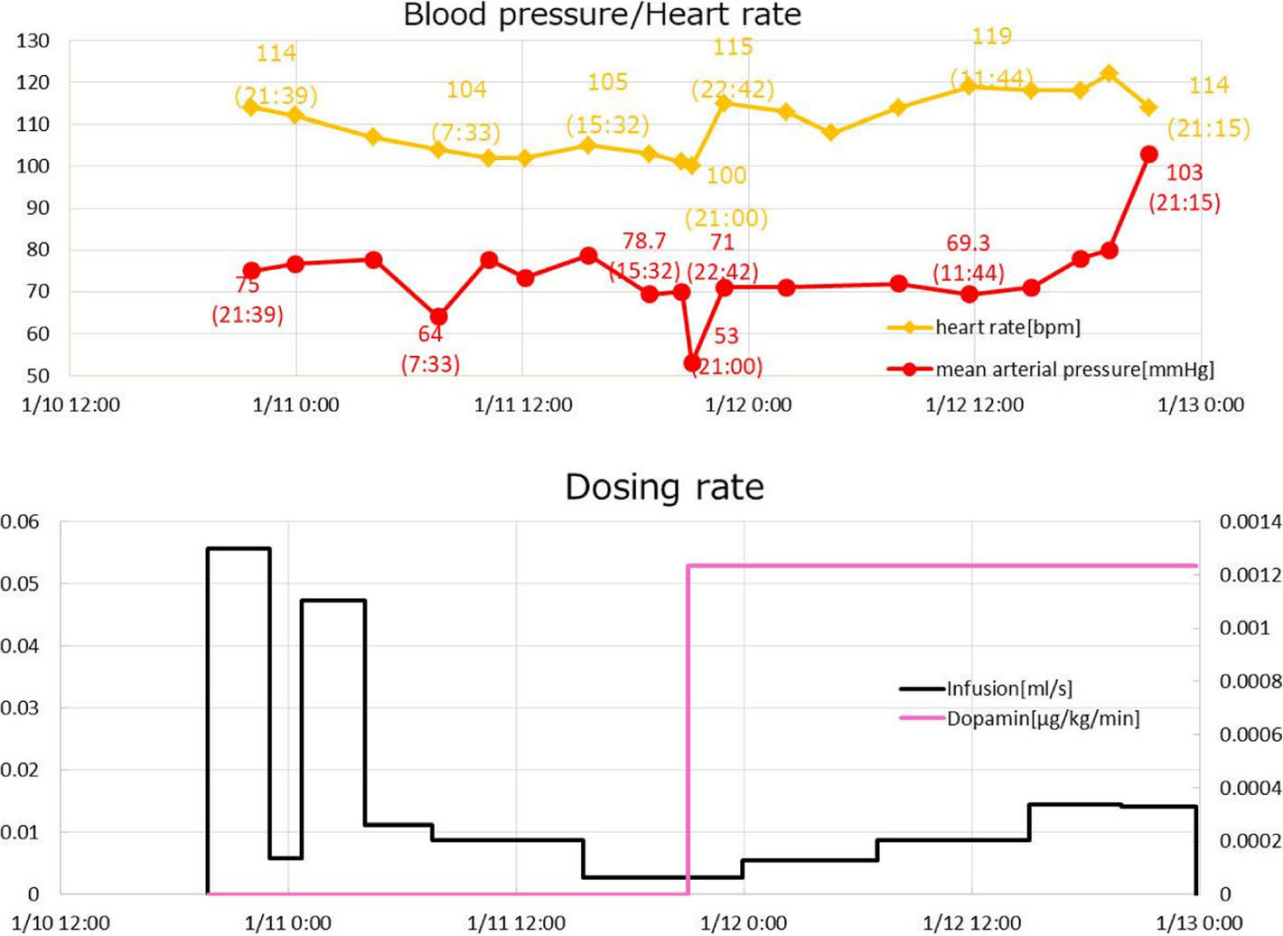
Clinical data for patient 2

**Fig. 11:**
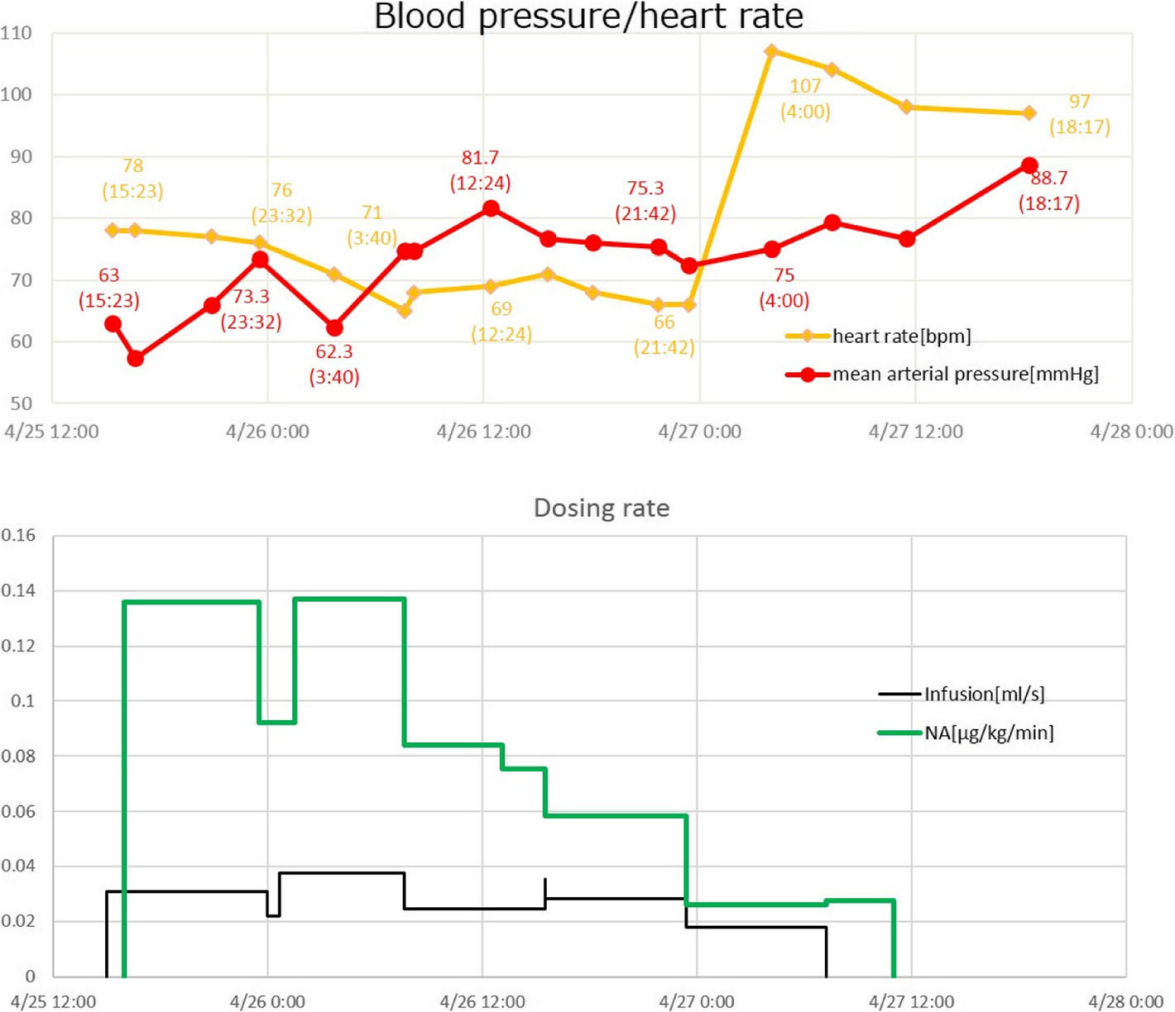
Clinical data for patient 3

For model validation, we used blood pressure and heart rate time courses, which were fundamental state variables for tracking disease progression and therapy. In Figs. 9, 10, 11, the time courses of blood pressure and heart rate records are shown with the infusion and drug administration records of each patient. The time according to the records is shown on the horizontal axis. The severity of infection was set as moderate for all cases.

**Fig. 12:**
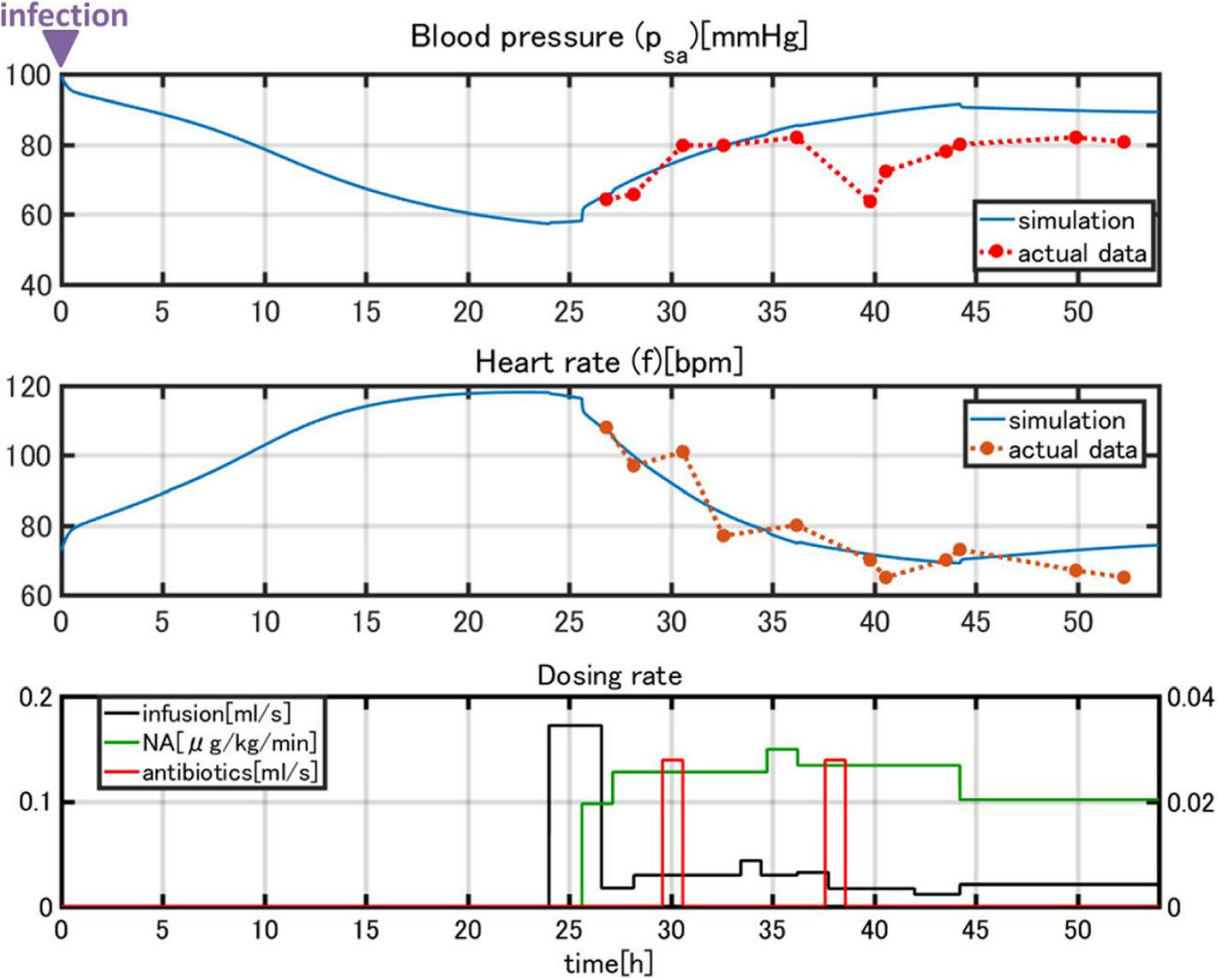
Comparison of the simulation results and clinical data for patient 1

We observed marked different time courses of sepsis progression among the three patients. We must adjust the parameters of the model to reproduce the data for each patient. We performed simulations to check whether the model parameters could be adjusted to fit the computational results to the clinical data. Parameters were adjusted starting with the model constructed for a *standard patient* in the first step of validation. Figure 12 compares the simulation results and clinical data for patient 1. The blood pressure (MAP) and heart rate computed by our model fit the clinical data well.

A sudden drop in blood pressure occurred 17 h after treatment began, which the model did not reproduce.

Normally, a drop in blood pressure is associated with an elevated heart rate according to the baroreflex response. However, in this case, the patient’s heart rate also dropped. Because this patient was a heavy habitual alcohol drinker (ca. 150 g/day), we thought a heart dysfunction was induced during septic shock. Thus, we imposed external noise *f*_*noise*_ on heart rate *f* given in equation (3) as

37 $$ f\to f+{f}_{noise} $$

**Fig. 13:**
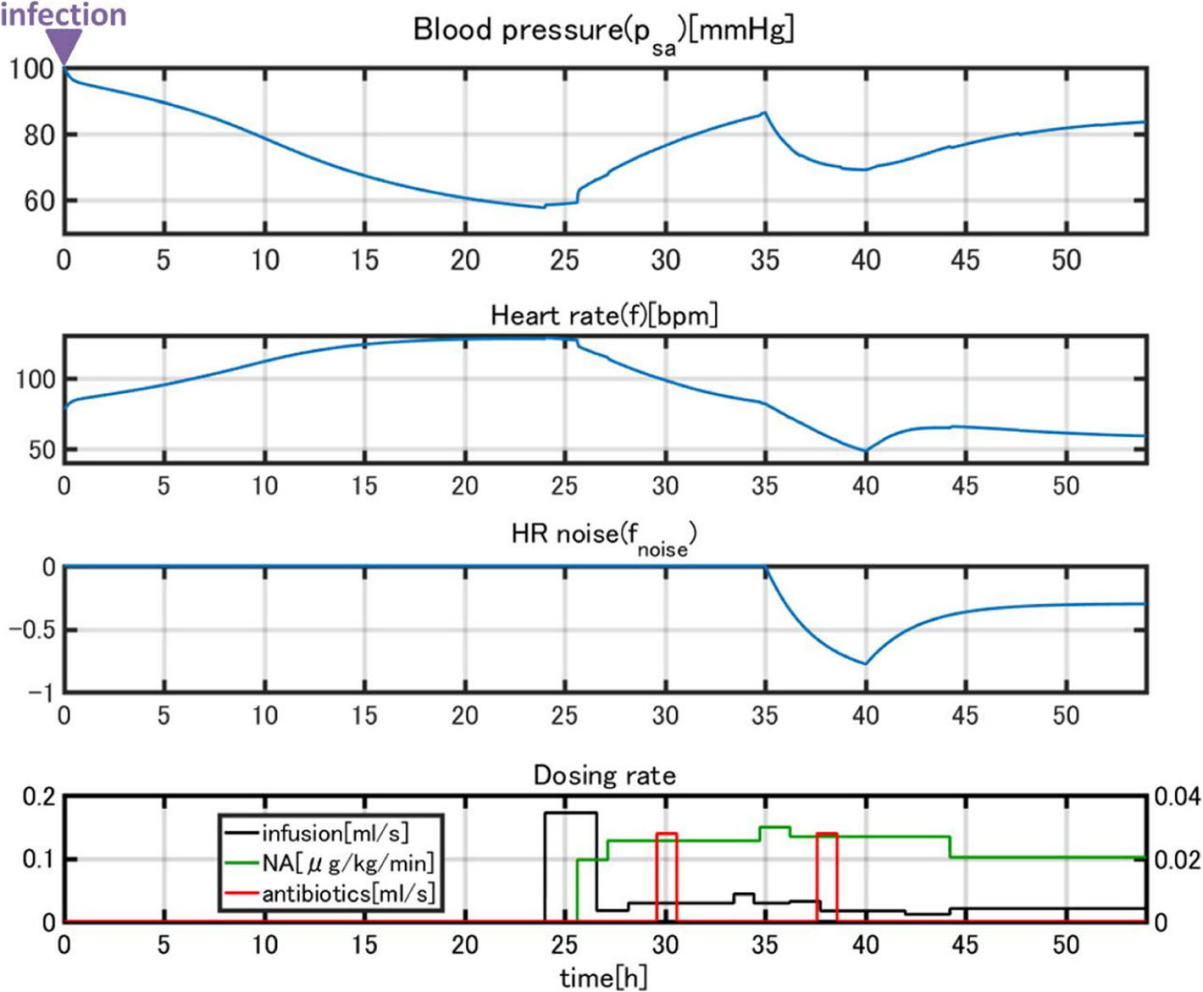
Simulation results after *f*_*noise*_ was introduced

**Fig. 14:**
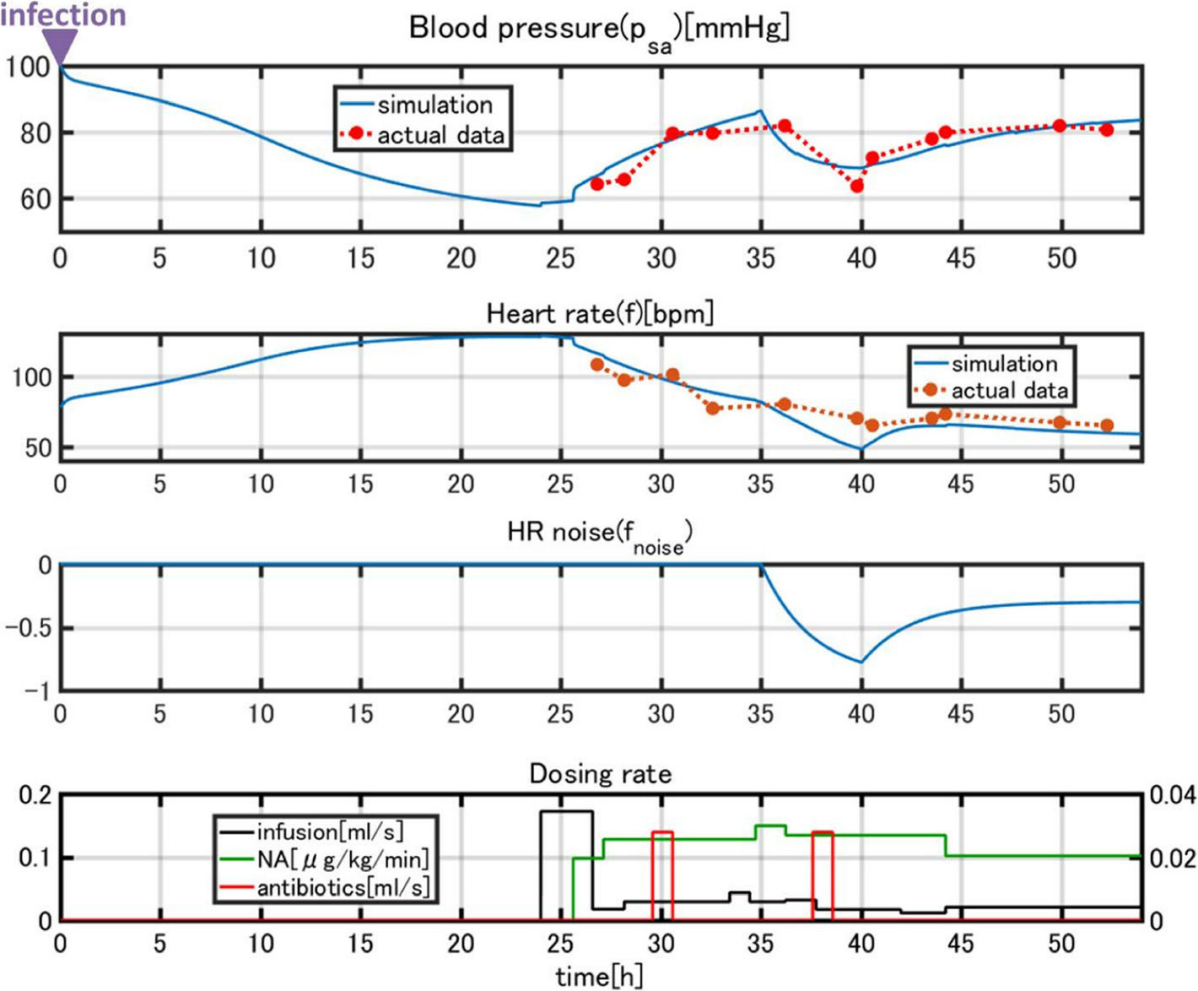
Comparison of the simulated and clinical data after *f*_*noise*_ was introduced

*f*_*noise*_ is shown in Fig. 13. We also assumed that a sudden reduction of arterial baroreceptor gain *q*_*r*_ in equation (3) occurred. The simulation results incorporating these events are shown in Fig. 13. The results reproduce the sudden drop in heart rate and the effect on blood pressure, as well as the recovery process (Fig. 14). Usually, disease progression is affected by many unexpected factors that cannot be represented in a model. However, a model can explain unexpected events when reasonable assumptions are made. In this case, our simulation was validated due to the close link between cardiac dysfunction and sepsis [35].

**Fig. 15:**
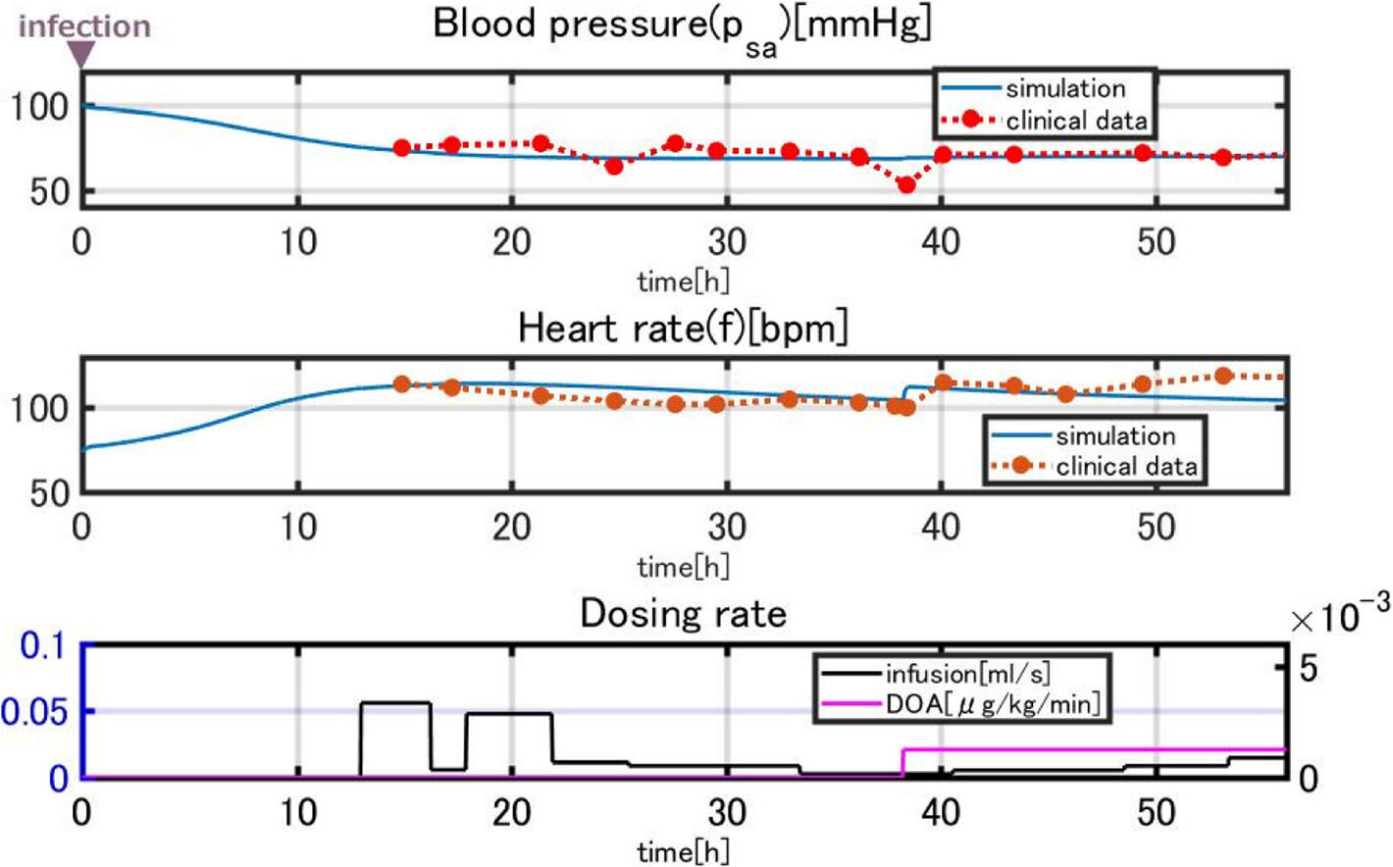
Comparison of the simulation results and clinical data for patient 2

Figure 15 compares the simulation results and clinical data for patient 2.

This patient was stable with low blood pressure and high heart rate when given infusion therapy. The administration of dopamine contributed to the elevation of the heart rate, which was reproduced in the simulation.

**Fig. 16:**
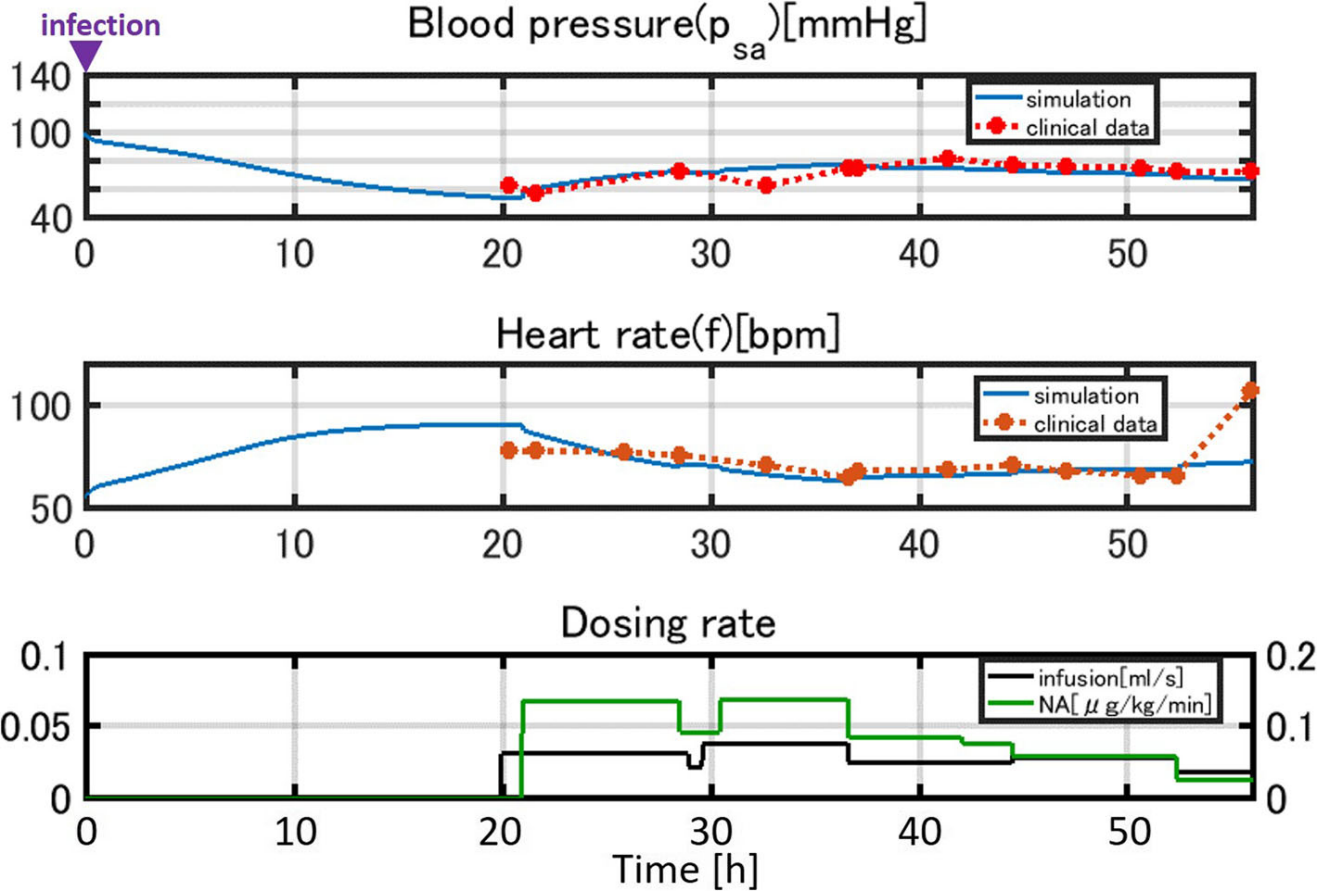
Comparison of the simulation results and clinical data for patient 3

Figure 16 compares the simulation results and clinical data for patient 3.

The gradual recovery of the blood pressure and the associated normalization of the heart rate due to infusion and noradrenaline administration are closely reproduced by the simulation.

The parameter tuning for fitting data was done by updating several parameters of the basic model used in the first step of the qualitative validation. Table 2 lists the parameters changed for accommodating the individual patients. Among 12 parameters, five are related to the vessel resistance. Remarkably, only a handful of parameters need to be adjusted for accommodating differences among patients, and also the differences among the parameter values are not large.

**Table 2:** Patients parameter information

Parameter	Standard Patient	Patient1	Patient2	Patient3
Normal value of heart rate *f*(*f*_0_)	1.2beat/s	1.2beat/s	1.25beat/s	0.9beat/s
Normal value of arteriovascular radius *r*(*r*_0_)	3.0*μ*m	3.0*μ*m	2.85*μ*m	2.9*μ*m
Initial total blood Volume(*V*_0_)	3150ml	5300ml	3150ml	5200ml
Severity(*k*_*pg*_)	-	0.55 /h	0.7 /h	0.6 /h
Rate of vessel radiusr dilation(*k*_*EX*_)	40	50	35	44
Compliance of sa(*C*_*sa*_)	4ml/mmHg	4ml/mmHg	2.8ml/mmHg	2.8ml/mmHg
Resistance of the systemic circulation upstream of arteriolar capillaries(*R*_*s*2_)	0.2247mmHg*∙* s/ml	0.2247mmHg*∙* s/ml	0.2247mmHg*∙* s/ml	0.2472mmHg*∙* s/ml
Resistance of the systemic circulation downstream of arteriolar capillaries(*R*_*s*3_)	0.1124mmHg*∙* s/ml	0.1124mmHg*∙* s/ml	0.1124mmHg*∙* s/ml	0.1236mmHg*∙* s/ml
Resistance of sv(*R*_*sv*_)	0.011mmHg*∙* s/ml	0.011mmHg*∙* s/ml	0.011mmHg*∙* s/ml	0.012mmHg*∙* s/ml
Resistance of pa(*R*_*pa*_)	0.1124mmHg*∙* s/ml	0.1124mmHg*∙* s/ml	0.1124mmHg*∙* s/ml	0.1236mmHg*∙* s/ml
Resistance of pv(*R*_*pv*_)	0.0056mmHg*∙* s/ml	0.0056mmHg*∙* s/ml	0.0056mmHg*∙* s/ml	0.0062mmHg*∙* s/ml
Rate at which activated phagocytes(N*) consume pathogen(*k*_*pn*_)	1.8 /N*-units/h	1.8 /N*-units/h	1.8 /N*-units/h	1.26/N*-units/h

*The table indicates the differences of the parameters among the three patients. The confidence intervals of the parameters are omitted in the table

An important parameter that is not listed in Table 2 is the time interval between the time of infection and the start of treatment. At the start of the simulation, the initial value of pathogen P is set to be positive. This implies that the starting time of the simulation is the time of infection. We must decide when treatment is started (when the initial data was obtained) based on the goodness of fit between data and simulations. This tuning is one-dimensional and was not difficult. It conveys, however, valuable information about when the patient was infected before hospitalization.

## Discussion

The first part of the simulation showed that our model captures the fundamental dynamics of sepsis progression and the effects of therapy. The severity of infection could be represented by the growth rate *k*_*pg*_ of the pathogen in the immune model in equation (19). We considered mild, moderate, and severe infections, and treatment with nothing, saline infusion, and saline infusion plus medicine to identify the level of difficulty of recovery. The results are shown in Fig. 7 and summarized in Table 3.

**Table 3:** Severity and intensity of treatment

	Mild	Moderate	Severe
No treatment	○	×	×
Infusion	○	○	×
Infusion and medicine	○	○	○

○: recovered ×: not recovered

The results show that the severity of the infection matched the intensity of treatment required for recovery.

Many sepsis patients have underlying diseases; thus, we used heart failure as an example of an underlying disease that can be modeled by changing some parameters. We showed that the patient with heart failure does not recover from even a mild infection without treatment, which is consistent with clinical experience. These simulations show that our model captures the essential features of sepsis and that the interactions quantified in our model among the immune, cardiovascular, and nervous system submodels are justified, at least qualitatively.

In the second part of the simulation, we validated our model to fit clinical data of three sepsis patients (Figs. 9–11). As stated in the Background section, the progression of sepsis differs among patients, and the symptoms are also different. Although it is necessary to customize the models for each patient by choosing appropriate model parameters, there are a large number of parameters making customization appear difficult. However, as was shown in Table 3, the number of parameters tuned for fitting was not very many and most parameters were unchanged from the general model of a standard patient used for the first part of simulations (Figs. 7 and 8). The most obvious differences were body weight and sex, which affect the total blood volume. Age differences were taken into account by choosing the vessel flexibility and radius, and the strength of the sympathetic nerve activity was tuned slightly to accommodate the data. They are natural, easy, and reasonable customizations for individuality.

Heart failure is included as an underlying disease in patient 1. The sudden drop in blood pressure is accommodated by a sudden drop in heart rate, which is usually a symptom of heart failure. This suggests the possibility that the model can explain sudden events occuring during the course of treatment. For patient 3, we ignored diabetes as an underlying disease, but still obtained a good fit between the model and data.

The most finely balanced and important parameter for fitting the model to real data is the time of infection or the starting time of the simulation. Typically, a patient has already been infected when admitted to the hospital and does not know when they were infected. As discussed in the Method sections, we could estimate the time of infection through a one-dimensional search. Estimating the time of infection with the model by finding the most appropriate initial time of simulation gives valuable information for determining the treatment strategy for a patient. This is an important benefit of disease modeling.

Because sepsis is a serious disease that affects almost all parts of the body, it may lead to other subsequent diseases, which we have not incorporated in the model. However, even when an unexpected physical event occurred, the model could identify the cause. Patient 1 had a sudden drop in blood pressure during treatment (Fig. 12), which was explained by a heart attack (Fig. 14). Although this was an estimate, it could provide valuable information for medical staff.

## Conclusion

We have constructed a simple mathematical model of septic shock to represent and predict disease progression and the effects of treatments. The model combined the cardiovascular and immune systems through the effects of inflammation, which are represented by increases in vessel permeability, vasodilation, and stroke volume reduction. We assumed the following three effects of sympathetic nerve activity responding to severe hypotension caused by infection: elevated heart rate, increase in vessel resistance, and decrease in blood volume unloading. We also introduced the fatigue effect of the sympathetic nervous system. The weaker effects of drugs in sepsis patients were also considered.

We demonstrate that our model is a reasonable model of septic shock and represents the therapeutic effects of treatment through in silico experiments. We also show the reduced therapeutic effects in patients with sepsis who have underlying heart failure.

We validated our model based on the clinical data of three sepsis patients and showed that the model reproduced the treatment course. Moreover, the model reproduced sudden physiological events in patients.

Although our model represents specific aspects of septic shock, which is complex and involves almost every organ in diverse ways, we show that we can construct a model that captures the essential features of this disease. We discuss the potential of the model to help with clinical decision making and promoting a deeper understanding of sepsis.

Because the model contained a number of parameters that must be set for simulations, the difficulty in determining appropriate numerical values has been identified as one of the main barriers to using mathematical models in medicine. The customization of models for individual patients is an additional difficulty. However, we found that a standard patient model can be constructed based on the existing physiological, medical, and pharmacological knowledge, as described in the first part of the Results section, although some parameters had to be taken from experimental data on animals. We were able to customize the standard patient model for three patients based on their age, sex, weight, and underlying disease, by tuning only several parameters of the standard patient model. The simulations well reproduced the data.

Our results suggest that disease modeling could help medical staff predict the patient’s condition and establish a clinical strategy for recovery. The possibility of estimating the infection time before treatments start is another benefit of the disease model.

The disease model extracts knowledge about human physiology relevant to the target disease, and the model can be customized by selecting relatively small number of parameters. We consider that the general and individual data are accommodated well in the model and that their integration can bring great benefits to clinical practice. We hope that our model will play a role in guiding practitioners toward model-based therapies. To achieve this goal, our model must be more reliable and versatile, and must be validated using a larger amount of clinical data, which we intend to tackle in the next step of our research.

## Appendix

**Table 4:** Cardio vascular system model (Ursino model) parameters

Parameter	Value	Description	Source
*C*_*sa*_	4ml/mmHg	Compliance of sa	[16]
*C*_*sv*_	111.11ml/mmHg	Compliance of sv	[16]
*C*_*ra*_	31.25ml/mmHg	Compliance of ra	[16]
*C*_*pa*_	6.56ml/mmHg	Compliance of pa	[16]
*C*_*pv*_	25.37ml/mmHg	Compliance of pv	[16]
*C*_*la*_	19.23ml/mmmHg	Compliance of la	[16]
*R*_*s2*_	0.2247mmHg∙ s/ml	Hydraulic resistance of the systemic circulation upstream of arteriolar capillaries	[16]
*R*_*s3*_	0.1124mmHg∙ s/ml	Hydraulic resistance of the systemic circulation downstream of arteriolar capillaries	[16]
*R*_*sv*_	0.011mmHg∙ s/ml	Hydraulic resistance of sv	[16]
*R*_*pa*_	0.1124mmHg∙ s/ml	Hydraulic resistance of pa	[16]
*R*_*pv*_	0.0056mmHg∙ s/ml	Hydraulic resistance of pv	[16]
*L*_*a*_	0.01ml/mmHg/s	Permeability coefficient of arterial capillaries	[16]
*L*_*v*_	0.062ml/mmHg/s	Permeability coefficient of venular capillaries	[16]
*V*_*usa*_	611.3ml	Unstressed volume of sa	[16]
*V*_*ura*_	25ml	Unstressed volume of ra	[16]
*V*_*upa*_	124ml	Unstressed volume of pa	[16]
*V*_*upv*_	120ml	Unstressed volume of pv	[16]
*V*_*ula*_	25ml	Unstressed volume of la	[16]
*k*_*l*_	20ml/mmHg	Slope of the stroke volume versus the atrial pressure relationship for the left heart	[16]
*k*_*r*_	34.028ml/mmHg	Slope of the stroke volume versus the atrial pressure relationship for the right heart	[16]
*p*_*la0*_	2.8mmHg	x -axis intercept of the stroke volume versus atrial pressure relationship for the left heart	[16]
*p*_*ra0*_	1.82mmHg	x -axis intercept of the stroke volume versus atrial pressure relationship for the right heart	[16]
*V*_*n*_	5300ml	Total blood volume in the basal condition	[16]
*V*_*rc*_	1300ml	Red blood cell volume	[16]
*p*_*san*_	100mmHg	Intravascular pressure in the sa in the basal condition	[16]
*p*_*svn*_	5mmHg	Intravascular pressure in the sv in the basal condition	[16]
*p*_*ran*_	4mmHg	Intravascular pressure in the ra in the basal condition	[16]
*p*_*pan*_	17mmHg	Intravascular pressure in the pa in the basal condition	[16]
*p*_*pvn*_	7mmHg	Intravascular pressure in the pv in the basal condition	[16]
*p*_*lan*_	6.5mmHg	Intravascular pressure in the la in the basal condition	[16]
*k*_*Na*_	25ml/s	Mass transfer coefficient of the cellular membrane for sodium	[16]
*β*_*Na*_	0.0704	Mass transfer coefficient of the cellular membrane for sodium	[16]
*k*_*K*_	6.67 ∙ 10^−2^ml/s	Mass transfer coefficient of the cellular membrane for potassium	[16]
*β*_*K*_	28.2	Mass transfer coefficient of the cellular membrane for potassium	[16]
*k*_*U*_	13ml/s	Mass transfer coefficient of the cellular membrane for urea	[16]
*β*_*U*_	1	Mass transfer coefficient of the cellular membrane for urea	[16]
*k*_*f*_	4 ∙ 10^−3^*L*^2^/s/mmol	Water exchange coefficient of the cellular membrane	[16]
*M*_*eq,ic*_	150mmol	Amount of other osmotically efficient solutes in the intracellular compartment	[16]
*M*_*eq,ex*_	150mmol	Amount of other osmotically efficient solutes in the extracellular compartment	[16]
*E*_*is*_	24.5mmHg/L	Interstitial space elastance	[16]
*V*_*isn*_	11L	Basal volume of the interstitial fluid	[16]
*V*_*icn*_	25L	Basal volume of the intracellular fluid	[16]
*c*_*p,pin*_	7.4g/dl	Basal protein concentration in the plasma	[16]
*c*_*p,isn*_	1.37g/dl	Basal protein concentration in the interstitial fluid	[16]
*M*_*k,ic*_(0)	3535mEq	Initial amount of potassium in the intracellular fluid	[16]
*Mk,ex*(0)	75mEq	Initial amount of potassium in the extracellular fluid	[16]
*M*_*Na,ic*_(0)	250mEq	Initial amount of sodium in the intracellular fluid	[16]
*M*_*Na,ex*_(0)	2130mEq	Initial amount of sodium in the intracellular fluid	[16]
*M*_*U,ic*_(0)	2130mEq	Initial amount of urea in the intracellular fluid	[16]
*M*_*U,ex*_(0)	375mmol	Initial amount of urea in the extracellular fluid	[16]
*Q*_*F*_	0.2083ml/s	Ultrafiltration rate of the replacement fluid	[16]
*Q*_*inf*_	0ml/s	Ultrafiltration rate of the replacement fluid	[16]
*c*_*Na,d*_	142mEq/L	Ultrafiltration rate of the replacement fluid	[16]
*c*_*Kd*_	62mEq/L	Ultrafiltration rate of the replacement fluid	[16]
*c*_*Ud*_	0	Concentration of urea in the dialysate	[16]
*F*_*p*_	0.94	Plasma fractions	[16]
*F*_*R*_	0.72	Red blood cell water fractions	[16]
*γ*_*U*_	1	Fraction of red blood cell water that participates in the transfer through the dialyzer	[16]
*R*_*DU*_	1	Donnan ratio for urea in red cells	[16]
*γ*_*Na*_	0	Fraction of red blood cell water that participates in the transfer through the dialyzer	[16]
*γ*_*K*_	0	Fraction of red blood cell water that participates in the transfer through the dialyzer	[16]
*Q*_*B*_	3ml/s	Bulk blood flow through the dialyzer	[16]
*D*_*Na*_	2.67ml/s	Dialysance (or clearance) of sodium	[16]
*D*_*K*_	2.67ml/s	Dialysance (or clearance) of potassium	[16]
*D*_*U*_	2.67ml/s	Dialysance (or clearance) of urea	[16]
*σ*_*Rn*_	0.7303mmHg∙ s/ml	Basal value of the sigmodideal static characteristic for the mechanism of systemic resistance control	[16]
Δ_*σR*_	1.4mmHg∙ s/ml	Amplitude of the sigmodideal static characteristic for the mechanism of systemic resistance control	[16]
*τ*_*R*_	6s	Time constant of the mechanism of systemic resistance control	[16]
*G*_*aR*_	0.02/mmHg	Central gain of the arterial controls for the mechanism of systemic resistance control	[16]
*G*_*cR*_	0.7/mmHg	Central gain of the cardiopulmonary controls for the mechanism of systemic resistance control	[16]
*σ*_*V n*_	2900ml	Basal value of the sigmodideal static characteristic for the mechanism of venous unstressed volume control	[16]
Δ_*σV*_	500ml	Amplitude of the sigmodideal static characteristic for the mechanism of venous unstressed volume control	[16]
*τ*_*V*_	20s	Time constant of the mechanism of venous unstressed volume control	[16]
*G*_*aV*_	10.8/mmHg	Central gain of the arterial controls for the mechanism of venous unstressed volume control	[16]
*G*_*cV*_	417/mmHg	Central gain of the cardiopulmonary controls for the mechanism of venous unstressed volume control	[16]
*σ*_*Tn*_	0.833s	Basal value of the sigmodideal static characteristic for the mechanism of heart period control	[16]
Δ_*σT*_	0.75s	Amplitude of the sigmodideal static characteristic for the mechanism of heart period control	[16]
*τ*_*T*_	2s	Time constant of the mechanism of heart period control	[16]
*G*_*aT*_	0.015/mmHg	Central gain of the arterial controls for the mechanism of heart period control	[16]
*G*_*cT*_	0/mmHg	Central gain of the cardiopulmonary controls for the mechanism of heart period control	[16]
*p*_*lat*_	4.5mmHg	Threshold value of left atrial pressure for activation of the sympathoinhibitory mechanism	[16]
*G*_*σ*_	4.5mmHg	Gain constant of the sympathoinhibitory mechanism	[16]
*τ*_*σ*_	120s	Time constant of the sympathoinhibitory mechanism	[16]
*τ*_*ϵ*_	2700s(acetate dialysis only)	Time constant of the acetate effect upon peripheral vessels	[16]

**Table 5:** Nervous system model parameters

Parameter	Value	Description	Sources
*a*_*f*_,max	1.3175	Maximum value of sympathetic nerve activity *a*_*f*_	Estimated
*a*_*r*_,max	8.6993	Maximum value of sympathetic nerve activity *a*_*r*_	Estimated
*a*_*V*_,max	0.9443	Maximum value of sympathetic nerve activity *a*_*V*_	Estimated
*a*_*f*_,min	0.1896	Minimum value of sympathetic nerve activity *a*_*f*_	Estimated
*a*_*r*_,min	2.6996	Minimum value of sympathetic nerve activity *a*_*r*_	Estimated
*a*_*V*_,min	0.8206	Minimum value of sympathetic nerve activity *a*_*V*_	Estimated
*k*_*γ*_	3.0 × 10^−5^	Sympathetic nerve effect coefficient for*γ*	Estimated
*a*_*f*0_	0.680	Sympathetic nervous activity for *f*_0_	Estimated
*a*_*r*0_	0.549	Sympathetic nervous activity for *r*_0_	Estimated
*a*_*V*0_	0.0935	Sympathetic nervous activity for *V*_0_	Estimated
*f*_0_	1.2beat/s	Normal value of heart rate *f*	Estimated by [16]
*R*_s1,0_	0.0113mmHg·s/ml	Normal value of arteriolar vascular resistance *R*_*s*1_	Estimated by [16]
*V*_*usv*,0_	2.9 × 10^−3^ml	Normal value of venous unstressed blood bolume *V*_*usv*_	Estimated by [16]
*r*_*0*_	3.0*μ*m	Normal value of arteriovascular radius *r*	Estimated by [17]
*k*_*f*_	1.01	Sympathetic nerve effect coefficient for *f*	Estimated
*k*_*r*_	8.2	Sympathetic nerve effect coefficient for *r*	Estimated
*k*_*V*_	1.0	Sympathetic nerve effect coefficient for *V*_*usv*_	Estimated

**Table 6:** Immune system model and antibiotics pharmacological effect model parameters

Parameter	Value	Description	Sources
*k*_*pm*_	0.6/M-units/h	Rate at which the non-specific local response(M) eliminates pathogen	[15]
*k*_*mp*_	0.01/P-units/h	Rate at which the non-specific local response is exhauseted by pathogen	[15]
*k*_*pn*_	1.8/*N**-units/h	Rate at which activated phagocytes(*N**) consume pathogen	[15]
*p*_*∞*_	20·10^6^/cc	Maximum pathogen population	[15]
*s*_*m*_	0.005/M-units/h	Source of non-specific local response	[15]
*μ*_*m*_	0.002/h	Decay rate for the non-specific local response	[15]
*s*_*nr*_	0.08N_R_-units/h	Source of resting phagocytes	[15]
*μ*_*nr*_	0.12/h	Decay rate of resting phagocytes	[15]
*μ*_*n*_	0.05/h	Decay rate of activated phagocytes	[15]
*k*_*dn*_	0.35D-units/h	Maximum rate of damage produced by activated phagocytes	[15]
*μ*_*d*_	0.02/h	Decay rate of damage	[15]
*k*_*cn*_	0.04/*C*_*A*_-units/h	Maximum production rate of the anti-inflammatory mediator	[15]
*k*_*cnd*_	48*N**-units/D-units	Relative effectiveness of activated phagocytes and damaged tissue in inducing production of the anti-inflammatory mediator	[15]
*s*_*c*_	0.0125*C*_*A*_-units/h	Source of the anti-inflammatory mediator	[15]
*μ*_*c*_	0.1/h	Decay rate of the anti-inflammatory meditor	[15]
*k*_*nn*_	0.01/*N**-units/h	Activation of resting phagocytes by previously activated phagocytes and their cytokines	[15]
*k*_*np*_	0.1/P-units/h	Activation of resting phagocytes(*N*_*R*_)by pathogen	[15]
*k*_*nd*_	0.02/D-units/h	Activation of resting phagocytes by damage(D)	[15]
*c*_*∞*_	0.28*C*_*A*_-units	Controls the strength of the anti-inflammatory mediator(*C*_*A*_)	[15]
*x*_*dn*_	0.06*N**-units	Determines level of activated phagocytes needed to bring damage production up to half its maximum	[15]
*k*	25	Coefficient for *S*_*rate*_	Estimated
*k*_0_	18	Coefficient for *S*_*d*_	Estimated
*k*_*D*_	0.35/h	Coefficient of *S*_*d*_ for *D*	Estimated
*S*_0_	73.8ml	Normal value of stroke volume *S*	Estimated by[32]
*k*_*a*_	1/h	Absorption rate constant from the gut compartment to the central compartment	[23]
*k*_*e*_	1/h	Elimination rate constant from the central compartment	[23]
*f*_*p*_	1	Free fraction in plasma	[23]
*V*_*d*_	1l/kg	Distribution volume	[23]
*ε*	0.01/h	Maximum kill rate constant	[13]
*γ*	0.5	Hill coefficient	[13]

**Table 7:** Blood pressure reduction model parameters

Parameter	Value	Description	Sources
*L*_*a*_,min	0.01ml/mmHg/s	Normal vascular permeability	[16]
*L*_*a*_,max	0.06ml/mmHg/s	Maximum vascular permeability	Estimated by[29][31]
*EC*_50, *La*_	0.22	Half maximal effective *N** value	Estimated
*slope*_*La*_	1.0	Maximum gradient value of *L*_*a*_	Estimated
*k*_*EX*_	50	Rate of vessel radiusr dilation	Estimated
*EC*_50, *EX*_	0.5	Half maximal effective EX value	Estimated
*slope*_*EX*_	3.0	Maximum gradient value of EX	Estimated
*k*_*s*_	1.0	Effect of inflammatory to stroke volume	Estimated

**Table 8:** Noradrenaline pharmacologival effect model parameters

Parameter	Value	Description	Source
*N A*_*eff*_ ,max	1.05	Maximum effect of noradrenaline	Estimated by [36]
*EC*_50, *N A*_	0.53kg/*μ*g	Half maximal effective NA value	Estimated by [36]
*slope*_*N A*_	1.09	Maximum gradient value of NA	Estimated
*G*_*N A,r*_	5.0	Interaction strength of noradrenaline to artery	Estimated by [25]
*G*_*N A,V*_	0.18	Interaction strength of noradrenaline to vein	Estimated by [25]
